# Sulfur Vacancy‐Engineered Co_9_S_8_‐Ni_3_S_4_ Heterostructure as a Hydrogen Spillover Catalyst for Efficient Alkaline Water Splitting

**DOI:** 10.1002/advs.202513610

**Published:** 2025-10-06

**Authors:** Shoushuang Huang, Tianyu Jin, Jie Zhang, Yong Jiang, Jiwen Hu, Hejingying Niu, Amene Naseri, Kajsa Uvdal, Zhangjun Hu, Jiujun Zhang

**Affiliations:** ^1^ School of Environmental and Chemical Engineering Shanghai University/Shanghai Key Laboratory of Atomic Control and Application of Inorganic 2D Supermaterials Shanghai 200444 China; ^2^ Division of Molecular Surface Physics & Nanoscience Department of Physics Chemistry and Biology Linköping University Linköping 58183 Sweden; ^3^ Agricultural Biotechnology Research Institute of Iran (ABRII) Agricultural Research, Education, and Extension Organization (AREEO) Karaj 3135933151 Iran; ^4^ College of Sciences/Institute for Sustainable Energy Shanghai University Shanghai 200444 China

**Keywords:** nickel sulfide, heterojunction, hydrogen spillover, electrocatalysis, water splitting

## Abstract

Developing highly efficient and robust catalysts based on earth‐abundant materials for electrochemical water splitting remains a great challenge. Herein, we report the synthesis of a well‐defined hydrogen spillover electrocatalyst, i.e., sulfur vacancy‐enriched Co_9_S_8_‐Ni_3_S_4_ hollow heterostructure, via a self‐sacrificial template strategy. The introduction of sulfur vacancies greatly decreases the work function of Ni_3_S_4_, thereby narrowing the work function difference (Δϕ) with Co_9_S_8_. The reduced electron density at their interface facilities the hydrogen species (H^*^) transfer to trigger hydrogen spillover. Density functional theory (DFT) calculations reveal that H_2_O molecules preferentially adsorb and dissociate at Co sites of Co_9_S_8_ to generate active H^*^ intermediates, which subsequently migrate to Ni sites of Ni_3_S_4_ domains for H_2_ formation. The hydrogen spillover mechanism is strongly supported by experimental characterizations, including pH‐dependent kinetics, in‐situ Raman and electrochemical impedance analysis. Benefiting from these synergistic effects, the titled catalyst exhibited excellent electrocatalytic activity for alkaline hydrogen evolution reaction, requiring only 83 mV to achieve 10 mA cm^2^, along with remarkable durability, showing no detectable degradation even at 1 A cm^2^ for 100 h. This work deepens the fundamental understanding of hydrogen spillover mechanism and offers a practical strategy for developing highly active and durable catalysts for water splitting.

## Graphical abstract

A sulfur vacancy‐engineered Co_9_S_8_‐Ni_3_S_4_ hollow heterostructure is rationally designed to trigger hydrogen spillover, where vacancy‐mediated interfacial modulation enables efficient H migration and accelerates HER kinetics.

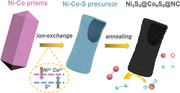

## 1 Introduction

The global pursuit of clean and renewable energy has spurred intensive efforts to develop advanced hydrogen production technologies. Among these, electrochemical water splitting has emerged as a viable and environmentally compatible route for green hydrogen generation.^[^ ^1^ ^]^ Typically, the overall water splitting process involves the hydrogen evolution reaction (HER) at the cathode and the oxygen evolution reaction (OER) at the anode, both of which suffer from sluggish kinetics and require efficient electrocatalysts to lower the overpotentials.^[^ ^2^, ^3^ ^]^ Although the noble‐metal‐based materials, such as Pt for HER and IrO_2_ or RuO_2_ for OER, have demonstrated outstanding catalytic activity and conductivity, the high cost and scarcity undoubtedly limit their widespread use.^[^ ^4^ ^]^ Up to now, significant efforts have been devoted to developing cost‐effective and stable earth‐abundant alternatives, but it remains a pressing challenge.

In recent years, transition metal compounds, such as oxides,^[^ ^2^, ^5^ ^]^ sulfides,^[^ ^6^, ^7^ ^]^ selenides,^[^ ^8^ ^]^ phosphides,^[^ ^9^, ^10^ ^]^ and carbides^[^ ^11^, ^12^ ^]^ have been extensively studied as HER or OER catalysts across a wide pH range. Particularly, cobalt and nickel‐based sulfides have attracted considerable attention as promising alternatives due to their abundant reserves, good conductivity, Pt‐like catalytic activity, and flexible valence states. For example, Sang et al.^[^ ^7^ ^]^ reported that the Mo and Fe co‐doped Ni_3_S_2_ nanoarray exhibited exceptional HER and OER catalytic activity in 1.0 m KOH, with overpotentials (η_10_) of only 59 and 186 mV required to reach 10 mA cm^−2^, respectively. Lyu et al. developed the CoS_2_‐Fe_x_Co_1‐x_S_2_ core–shell catalyst, which delivered superior OER and HER activity with low overpotentials of 235 and 342 mV at 100 mA cm^−2^, respectively.^[^ ^13^ ^]^ Du et al. demonstrated that the well‐designed CoS_1.097_/Ni_3_S_2_ catalyst on nickel foam exhibited outstanding catalytic activity for urea‐water splitting, operating at just 1.22 V to deliver a current density of 100 mA cm^−2^ for urea oxidation reaction (UOR), and at 1.27 and 1.57 V to reach 10 and 100 mA cm^−2^ respectively, in a UOR‖HER cell.^[^ ^14^ ^]^ Despite these encouraging advances, the Co and Ni‐based sulfides catalysts still have limitations of suboptimal hydrogen adsorption free energy (ΔG_H*_), insufficient active sites, and sluggish mass transport.

Up to now, various strategies have been employed to improve their intrinsic catalytic activity, including heteroatom doping,^[^ ^15^, ^16^, ^17^ ^]^ construction of heterostructure,^[^ ^9^, ^10^, ^18^ ^]^ and defect engineering.^[^ ^1^, ^19^ ^]^ Most recently, hydrogen spillover mechanism opens a new avenue for advancing HER electrocatalytic activity by enhancing hydrogen coverage at active sites and lowering kinetic barriers.^[^ ^20^, ^21^, ^22^, ^23^, ^24^, ^25^, ^26^, ^27^ ^]^ In these systems, H_2_O molecules are typically adsorbed and dissociated at the active metal sites (such as Ru site), generating active hydrogen H^*^ species that subsequently migrate to neighboring domains with more favorable hydrogen binding characteristics for H_2_ formation. Apparently, the spatial decoupling of H_2_O molecules dissociation and H^*^ adsorption/desorption allows each step to be proceeded under optimized local electronic structure, leading to more efficient utilization of catalytic active sites. Therefore, the HER kinetics can be significantly accelerated and exhibited better overall water splitting performance. For instance, Jiang et al. constructed a hydrogen spillover catalyst of NiSe_2_‐Ni_5_P_4_. The small work function difference (∆Φ) effectively mitigated interfacial charge accumulation and enabled smooth H^*^ transfer from NiSe_2_ to Ni_5_P_4_, which endowed the catalyst with excellent HER performance.^[^ ^22^ ^]^ Similarly, Xu et al. developed a dual‐site Co_n_‐Pt_1_@NPC catalyst, in which the Co nanoparticles served as water dissociation centers and delivered the H^*^ species to the isolated Pt atoms via a well‐defined spillover pathway, achieving markedly improved HER catalytic activity.^[^ ^21^ ^]^ Additionally, Li et al. employed an alloying strategy to reduce the ∆Φ between PtX (X = Ir, Pd, Co) alloys and CoP, thereby triggering hydrogen spillover for better HER performance.^[^ ^28^ ^]^ However, in many sulfide‐based heterostructures, the large mismatch of work function between different phase led to excessive charge localization at the interface, creating an energetic bottleneck for the migration of H^*^ and impending the hydrogen spillover. Therefore, tailoring the interfacial electronic structure, particularly by minimizing the ΔΦ of the building blocks, is critical to exploit the benefits of hydrogen spillover.

Among various strategies, defect engineering has proven to be an effective strategy for modulating the work function of electrocatalysts. By introducing the atomic‐scale defects such as vacancies, the local electronic structure can be greatly altered, leading to a redistribution of surface charge density and a shift in the Fermi level. These changes contribute to a reduced work function, thereby narrowing the energy difference between different components to facilitate the hydrogen spillover process. From previous study, the introduction of oxygen vacancies into Ru/NiMoO_4‐x_ heterojunction could effectively dilute the built‐in electric fields, thereby facilitating hydrogen spillover from NiMoO_4‐x_ to Ru with enhanced HER activity toward seawater splitting.^[^ ^25^ ^]^ Besides, the regulation of oxygen vacancy in the Ru/TiO_x_ composites effectively reduced the ∆Φ, which triggered hydrogen spillover to accelerate the HER process.^[^ ^23^ ^]^ Inspired by these studies, introducing sulfur vacancies into sulfide‐based catalysts is expected to offer comparable advantages, which are favorable for promoting the migration of H^*^ intermediates across the heterointerface and improving the intrinsic HER kinetics of catalysts. However, the impacts of sulfur vacancies on the hydrogen spillover behaviors for sulfide‐based HER catalysts has remained underexplored, and deserves in‐depth investigation.

Motivated by these insights, we designed and synthesizedsulfur vacancy‐enriched Co_9_S_8_‐Ni_3_S_4_ heterostructure as a hydrogen spillover catalyst by using a self‐sacrificial template method followed by controlled annealing treatment. The solid bimetallic Ni‐Co nanoprisms were first synthesized and subsequently treated with (NH_4_)_2_ solution in a mixed ethanol/water system. The chemical etching/ion‐exchange reactions induced the formation of hollow Ni‐Co‐S intermediates. Upon thermal annealing, the Ni‐Co‐S intermediates were transformed into Ni_3_S_4_ and Co_9_S_8_ phases, while the residual organics were converted into conductive nitrogen‐doped carbon (NC). Density functional theory (DFT) calculation results indicate that the introduction of sulfur vacancies decreases the work function of Ni_3_S_4_, thereby narrowing the work function difference (ΔΦ) between Ni_3_S_4_ and Co_9_S_8_, from from 0.61 to 0.28 eV, leading to charge redistribution and a weakened H* binding at the interface. This structural and electronic optimization enabled spatial separation of H_2_O dissociation and hydrogen evolution steps, with Co sites favoring H_2_O adsorption and dissociation, and Ni sites in Ni_3_S_4_ facilitating the H_2_ formation. As a sequence, the synergistic effects led to enhanced HER activity in alkaline media, along with excellent stability. Also, it exhibited excellent OER electrocatalytic activity with a low η_10_ of 265 mV in 1.0 m KOH. This work not only deepens the fundamental understanding of hydrogen spillover mechanis, but also offers a promising strategy for developing cost‐effective and durable catalysts for electrocatalysis.

## 2 Results and Discussion

### 2.1 Mechanistic Understanding of Hydrogen Spillover Via DFT

**Figure 1:**
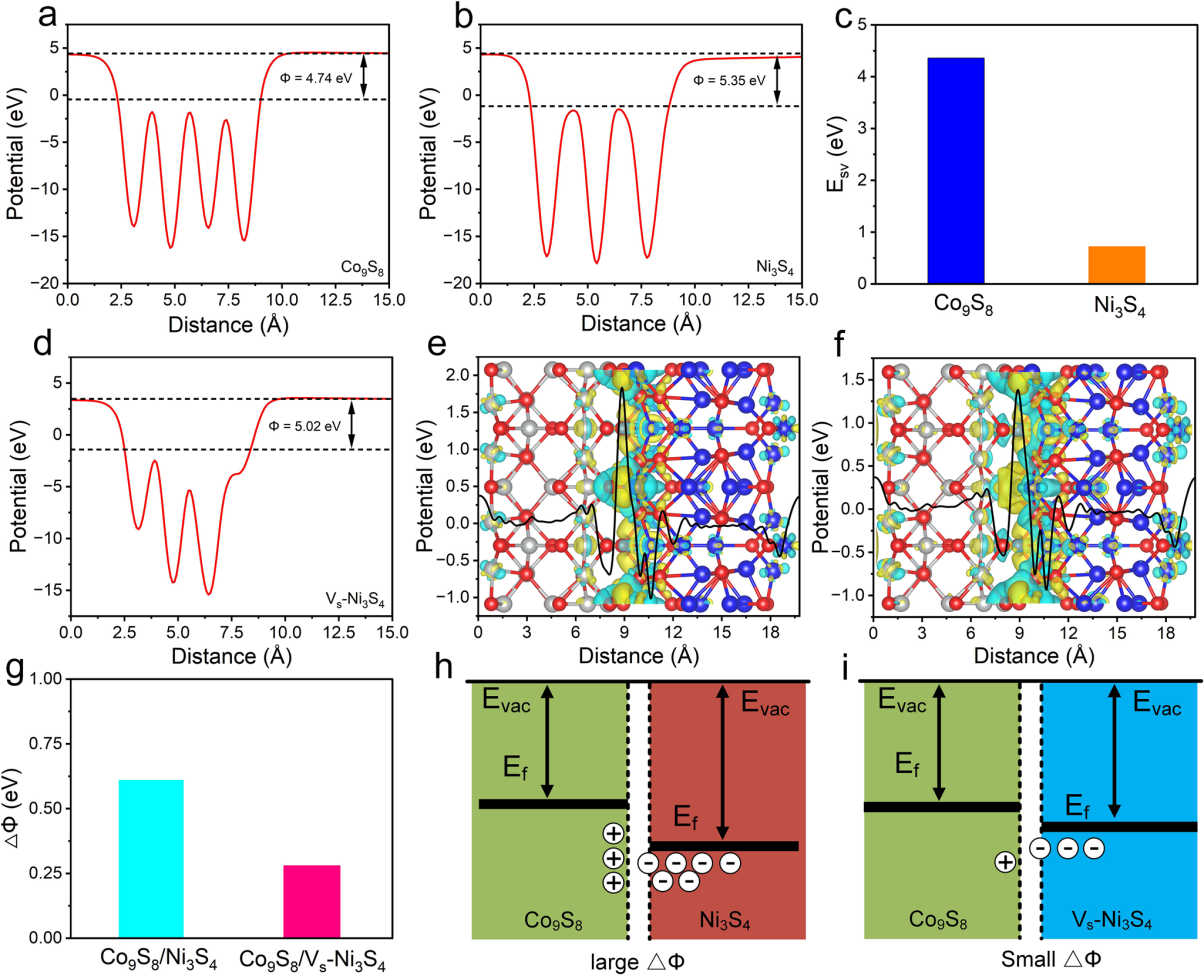
Work function of Ni_3_S_4_ (a), Co_9_S_8_ (b), and S_v_‐Ni_3_S_4_ (d). c) The summary of sulfur vacancy formation energy. Difference in charge density plot at the interface of Ni_3_S_4_‐Co_9_S_8_ (e) and Co_9_S_8_/V_s_‐Ni_3_S_4_ (f). g) Comparison of ΔΦ before and after sulfur vacancy introduction. Schematic illustrations of the electronic structure of Co_9_S_8_/Ni_3_S_4_ (h) and Co_9_S_8_/V_s_‐Ni_3_S_4_ (i).

For semiconductor heterojunctions, the relative work function (WF) of building blocks plays a critical role in determining the electron transfer direction and the band bending at the interface. Typically, electrons will flow from the semiconductor with a lower WF to the one with a higher WF until their Fermi levels alignment is achieved.^[^ ^29^, ^30^ ^]^ This interfacial charge redistribution not only determines the steady‐state electronic structure but also strongly influences the adsorption of intermediates and reaction kinetics during electrocatalysis.^[^ ^31^ ^]^ To explore the electronic structure of Ni_3_S_4_ and Co_9_S_8_ and the resulting interfacial electronic properties, DFT calculations were first conducted. As shown in **Figure** **1**a,b, the calculated WF for Co_9_S_8_ and Ni_3_S_4_ are 4.74 and 5.35 eV, respectively. Therefore, electrons will spontaneously transfer from Co_9_S_8_ to Ni_3_S_4_ after their combination to form a heterojunction, resulting in an electron‐rich Ni_3_S_4_ domain and an electron‐deficient Co_9_S_8_ region.^[^ ^32^ ^]^ The directional charge transfer will establish an internal electric field at the interface and induce significant electron accumulation, therefore strengthening the interactions between H* and interfacial active sites.^[^ ^33^ ^]^ Such a strong binding can promote the initial H_2_O adsorption and H^*^ formation, however, it simultaneously creates a kinetic barrier for hydrogen spillover, which impedes the subsequent migration of H^*^ species across the interface.

The sulfur vacancy formation energies (E_sv_) for Ni_3_S_4_ and Co_9_S_8_ were then calculated to understand how defect engineering modulates the interfacial electronic structure. As presented in Figure 1c, the Co_9_S_8_ exhibits a much higher E_sv_ of 4.36 eV compared to that of Ni_3_S_4_ (0.72 eV), indicating that the sulfur vacancies are energetically more favorable in the Ni_3_S_4_ domain. As we expected, the introduction of sulfur vacancies into Ni_3_S_4_ (V_s_‐Ni_3_S_4_) significantly reduces the WF, from 5.35 to 5.02 eV. To further explore the effects of sulfur vacancies on the interfacial charge behaviors, electron density difference mapping was computed. As shown in Figure 1d, the yellow and cyan regions represent electron accumulation and depletion, respectively. For the Co_9_S_8_/Ni_3_S_4_ heterostructure, a pronounced electron cloud is observed, indicating the strong interfacial charge transfer at the interface.^[^ ^28^ ^]^ In contrast, after introducing sulfur vacancies into Ni_3_S_4_, the interfacial electron accumulation in the Co_9_S_8_/V_s_‐Ni_3_S_4_ structure is diluted. This result is consistent with the reduced ΔΦ shown in Figure 1g, demonstrating that sulfur vacancies mitigate excessive interfacial electron transfer and promote a more balanced charge distribution. In this way, the interfacial electronic structure modulation weakens the binding of H^*^ intermediates, facilitates their migration across the interface, and enables a more efficient hydrogen spillover process.^[^ ^34^, ^35^ ^]^ The Bader charge analysis quantitatively supports this conclusion, with a reduced total interfacial charge transfer from 5.01 e (Co_9_S_8_/Ni_3_S_4_) to 4.22 e (Co_9_S_8_/V_s_‐Ni_3_S_4_) upon sulfur vacancy introduction. This result confirms that the sulfur vacancies disrupt the electron supply, allowing more electrons to be retained on the Co_9_S_8_ domins. Therefore, the dissociation of H_2_O and H^*^ formation preferentially occurs on Co_9_S_8_, while the H^*^ activation and subsequent hydrogen evolution are facilitated on the V_s_‐Ni_3_S_4_ domain (Figure 1h,i). This directional H^*^ transfer establishes a hydrogen spillover pathway, thus reducing thermodynamic and kinetic barriers to accelerates HER.

### 2.2 Physical and Chemical Structure Characterizations

**Figure 2:**
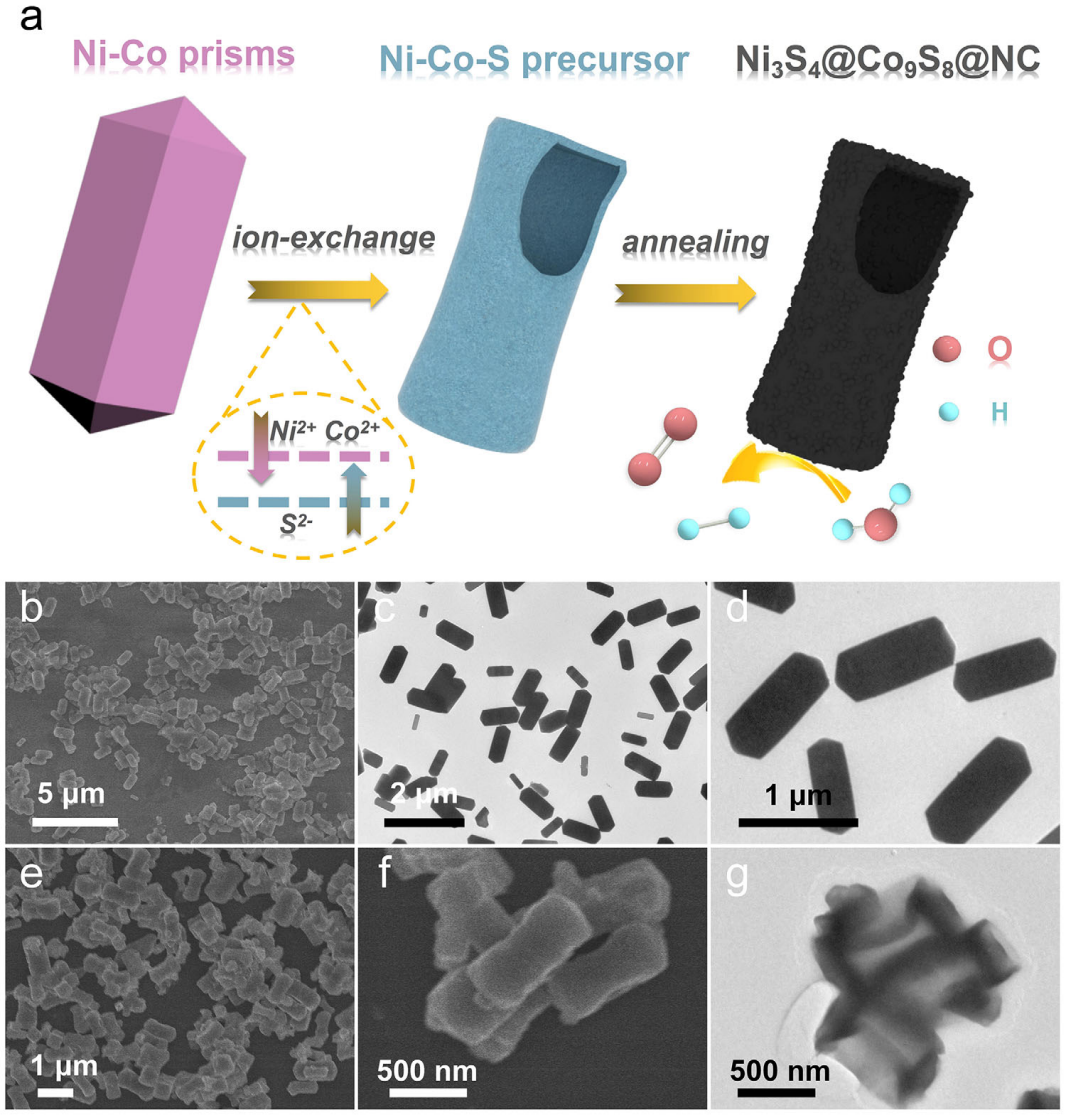
a) Schematic illustration of the synthesis of Ni_3_S_4_/Co_9_S_8_@NC catalyst. SEM (b, e, f) and TEM (c, d, g) images of the as‐synthesized Ni‐Co nanoprisms (b–d) and Ni‐Co‐S precursor (e–g).

Following the DFT calculations, we successfully synthesized the sulfur vacancy‐enriched Ni_3_S_4_/Co_9_S_8_ heterostructure via a simple self‐sacrificial template strategy, as illustrated in **Figure** **2**a. First, nearly monodispersed Ni‐Co nanoprisms were prepared as templates through a polyol‐mediated condensation reflux process. Subsequently, the resultant NiCo‐nanoprisms were immersed into a mixed ethanol/water solvent followed by adding certain amount of 15 wt.% (NH_4_)_2_S solution. Due to the hydrolysis of (NH_4_)_2_S in the solution, a high concentration of S_x_^2−^ and OH^−^ ions were generated, which induced the partial dissolution of Ni‐Co nanoprisms. The released Ni^2+^and Co^2+^ ions subsequently reacted with S_x_^2−^ ions to form an amorphous metal sulfide shell (named as Ni_x_Co_y_). As the outward diffusion of Ni^2+^ and Co^2+^ ions are faster than the inward migration of S^2−^ ions, leading to the voids accumulated within the core of NiCo‐nanoprism and finally yielding a well‐defined hollow structure.^[^ ^36^ ^]^ The subsequent annealing treatment under N_2_ atmosphere facilitated the crystallization of amorphous metal sulfide shell into Ni_3_S_4_ and Co_9_S_8_ phases. Meanwhile, the surface‐adsorbed PVP molecules were converted into an ultrathin N‐doped carbon layer. In this way, the Ni_3_S_4_/Co_9_S_8_ heterostructure encapsulated into carbon layer (Ni_3_S_4_/Co_9_S_8_@NC) was obtained, which exhibited enhanced surface area, optimized interfacial electronic structure, and efficient mass and charge transport pathways. All of these advantages contributed to its outstanding electrocatalytic activity for overall water splitting.

The morphological transformations from solid Co‐nanoprisms and Ni‐Co nanoprisms to hollow Co‐S and Ni‐Co‐S intermediates were investigated by scanning electron microscopy (SEM) and transmission electron microscopy (TEM). As shown in SEM images of Figure 2b, the Ni‐Co nanoprisms exhibited typical hexagonal prismatic geometries with a smooth surface. The average dimensions of Ni‐Co nanoprisms is measured to be 1.24 ± 0.3 µm in length and 0.63 ± 0.1 µm in width. The shape and size of the Co‐nanoprisms is similar to that of Ni‐Co nanoprisms (Figure S1, Supporting Information). The TEM images in Figure 2c,d confirms their solid structure with sharply defined edges. These edge regions are typically defect‐enriched, thereby providing energetically favorable sites for initiating chemical reactions.^[^ ^37^ ^]^ Consequently, an obvious structural reorganization was observed after the solvothermal treatment in the (NH_4_)_2_S solution. As seen in the SEM images of Figure 2e,f, the initial sharp ends of the nanoprisms became indistinct, and the central region exhibited noticeable bending, resulting in a biconcave, bone‐like shape with thicker ends and a constricted center. Additionally, the dimensions decreased with the length reduced by ≈22% to 0.95 ± 0.2 µm and the width reduced by ≈14% to 0.54 ± 0.1 µm. The hollow structure of the Ni‐Co‐S intermediate was further confirmed by TEM analysis. As shown in Figure 2g, all the particles exhibited well‐defined hollow cavities, with a shell thickness of ~50 nm. The morphology changes implied great mass redistribution and structural softening during the solvothermal process.

To elucidate the evolution of phase and crystal structure of the Ni‐Co‐S intermediates, X‐ray powder diffraction (XRD) measurements were conducted. The diffraction peaks of pristine Ni‐Co nanoprisms can be well indexed to nickel‐cobalt hydroxyacetate ((Ni, Co)_5_(OH)_2_(CH_3_COO)_8_·2H_2_O, JCPDS No. 22‐0582) (Figure S2a, Supporting Information). It was found that the XRD diffraction peaks of Ni‐Co‐S are similar to Ni‐Co nanoprism (Figure S2b, Supporting Information), and no diffraction peaks corresponding to Ni/Co‐based sulfides can be observed. This phenomenon can be attributed to the poor crystallinity of the as‐formed sulfides as the chemical transformation was performed at room‐temperature. The shape and phase evolution indicated the reaction proceeded via a partial anion exchange and surface reconstruction, rather than through a complete dissolution‐reprecipitation pathway.

It was found that the amount of (NH_4_)_2_S solution, reaction temperature, and reaction time played a crucial role in determining the formation and integrity of the hollow structure. As discussed earlier, the (NH_4_)_2_S serves both as a sulfur source and an etching agent. When only 0.1 mL was used, the sulfidation reaction was incomplete, and the resulting product retained a dense, solid structure due to insufficient etching (Figure S3, Supporting Information). On the other hand, increasing the amount to 1.0 mL led to excessive etching, which severely disrupted the structural framework and caused collapse of the template (Figure S4, Supporting Information). An optimized amount of 0.5 mL (NH_4_)_2_S was identified as suitable for promoting a uniform hollow structure with well‐preserved morphology. Additionally, the reaction temperature influences sulfidation kinetics and ions diffusion behaviors. The moderate room‐temperature enabled a balanced outward diffusion of cations and inward migration of sulfur species, thereby promoting the formation of hollow structures via the Kirkendall effect. However, a higher temperature of 50 °C induced overly rapid reactions, leading to structural instability and deformation (Figure S5, Supporting Information). Similarly, the reaction time is another critical parameter. A shorter duration limited the extent of sulfidation, whereas a prolonged time of 2 h resulted in particle aggregation and collapse of the nanoprism templates (Figure S6, Supporting Information). The above control experiments clearly demonstrated that the precise control over reaction rate was essential for constructing the hollow nanostructure.

**Figure 3:**
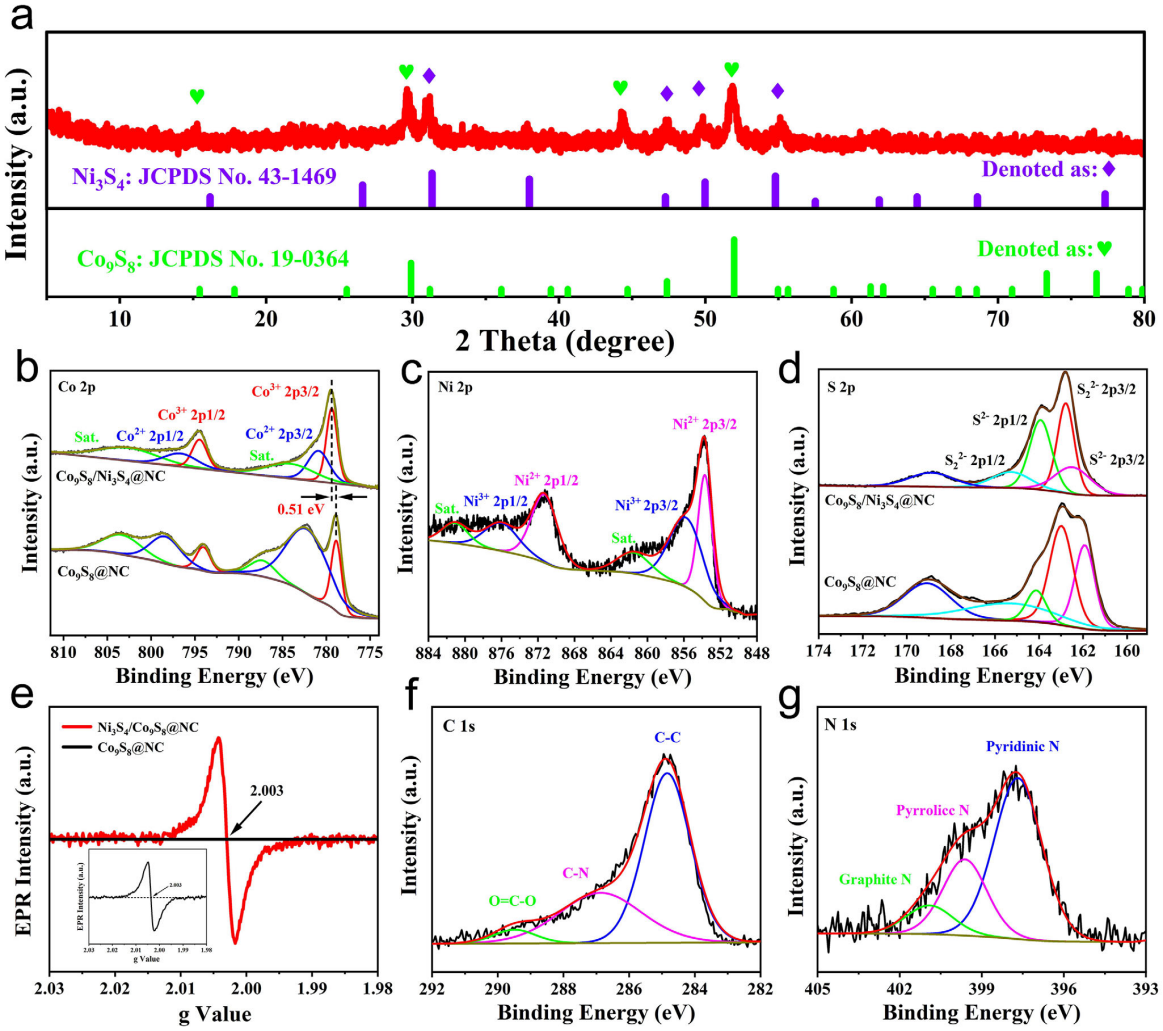
XRD patterns (a) and high‐resolution XPS spectra of Co 2p (b), Ni 2p (c), S 2p (d), C 1s (f) and N 1s (g) for the as‐synthesized Co_9_S_8_@NC and Ni_3_S_4_/Co_9_S_8_@NC samples. e) ESR spectra of the Ni_3_S_4_/Co_9_S_8_@NC catalyst.

Although the resulting Ni‐Co‐S intermediate exhibited a relatively large specific surface area that might expose numerous electrocatalytic active sites, its intrinsic poor conductivity and disordered atomic structure will greatly impede the charge transport during the electrochemical process, ultimately limiting its HER and OER performance. To overcome this drawback and further improve their electrocatalytic performance, the Ni‐Co‐S and Co‐S intermediates were subjected to thermal annealing at 350 °C for 2 h under an inert N_2_ atmosphere. The corresponding XRD patterns of the Ni_3_S_4_/Co_9_S_8_@NC catalyst was presented in **Figure** **3**a. The diffraction peaks observed at 16.2°, 26.6°, 31.3°, 38.0°, 47.3°, 50.0°, and 54.8° can be well indexed to the (111), (022), (113), (004), (224), (115), and (044) planes of Ni_3_S_4_ (JCPDS No. 43‐1469), respectively. In addition, the peaks located at 29.9°, 47.4°, 52.0°, and 73.3° correspond to the (311), (511), (440), and (731) planes of Co_9_S_8_ (JCPDS No. 19‐0364), indicating the coexistence of Ni_3_S_4_ and Co_9_S_8_ phases. The absence of diffraction peaks of nickel‐cobalt hydroxyacetate suggested that it was decomposed and converted into sulfides completely. The Co‐S precursor was also subjected to the same annealing process, and the resulting diffraction peaks can be indexed to Co_9_S_8_ (Figure S7, Supporting Information).

X‐ray photoelectron spectroscopy (XPS) was subsequently performed to analyze the surface composition and chemical states of the Ni_3_S_4_/Co_9_S_8_@NC and Co_9_S_8_@NC catalysts. The survey spectrum of Ni_3_S_4_/Co_9_S_8_@NC confirms the coexistence of Ni, Co, S, N, and C elements in the catalyst (Figure S8, Supporting Information), indicating the successful integration of Ni/Co‐based sulfides and N‐doped carbon. In the high‐resolution Co 2p spectra, both samples exhibit two sets of spin‐orbit doublets corresponding to cobalt species with different valence states. Specifically, for Ni_3_S_4_/Co_9_S_8_@NC, the Co^3+^ 2p3/2 and 2p1/2 peaks were located at 779.47 and 794.47 eV, while the Co^2+^ 2p3/2 and 2p1/2 peaks appeared at 780.97 and 796.85 eV, respectively.^[^ ^38^ ^]^ The peaks at 784.45 and 803.17 eV can be attributed to satellites. Notably, the peaks of Ni_3_S_4_/Co_9_S_8_@NC show an upward shift of ≈0.51 eV compared to the Co_9_S_8_@NC sample, indicating a decrease of electron density around Co atoms.^[^ ^39^ ^]^ Additionally, the Co^3+^/Co^2+^ peak area ratio of Ni_3_S_4_/Co_9_S_8_@NC was much higher than that, suggesting an increased cobalt oxidation state, consistent with the interfacial electron redistribution revealed by DFT calculations. The Ni 2p spectrum of Ni_3_S_4_/Co_9_S_8_@NC displayed peaks at 853.7 and 871.4 eV corresponding to Ni^2+^, and the peaks at 856.2 and 875.4 eV corresponded to the Ni^3+^ species, accompanied by satellite features at 861.6 and 881.2 eV (Figure 3c).^[^ ^40^ ^]^ The S 2p spectrum displays S^2–^ peaks at 162.2 eV (2p3/2) and 163.8 eV (2p1/2), and peaks at 162.7 and 165.3 eV due to S_2_^2–^ and a broad feature at 168.7 eV corresponding to oxidized sulfur species (SO_x_^2–^) (Figure 3d). The relative peak area of the 2p1/2 increased from 50.12% in Co_9_S_8_@NC to 57.77% in Ni_3_S_4_/Co_9_S_8_@NC, indicating a higher concentration of sulfur vacancies.^[^ ^41^, ^42^ ^]^ This is further supported by the electron spin resonance (ESR) spectroscopy, where a strong signal at g ≈ 2.004 confirmed the presence of unpaired electrons associated with sulfur vacancies (Figure 3e). The ESR spectra of Co_9_S_8_@NC and Ni‐Co‐S precursor were also collected for comparison (Figure S9, Supporting Information). Strikingly, the ESR intensity of Ni_3_S_4_/Co_9_S_8_@NC is ≈6.7 × 10^4^ times higher than that of Co_9_S_8_@NC, indicating that the majority of sulfur vacancies are located in the Ni_3_S_4_ phase rather than Co_9_S_8_, which is in good agreement with the DFT results of E_sv_. The sulfur vacancies can modulate the local electronic environment, enhance water adsorption, and promote Volmer step during HER, contributing to reduced overpotentials and enhanced catalytic kinetics. The N 1s spectrum resolved three components of pyridinic N (397.8 eV), pyrrolic N (399.6 eV), and graphitic N (400.9 eV), implying the conversion of PVP into N‐doped carbon (Figure 3f).^[^ ^43^ ^]^ In the C 1s spectrum, the peaks at 284.8, 286.7, and 289.4 eV were assigned to C─C/C═C, C─N, and C─O bonds, respectively (Figure 3g).

**Figure 4:**
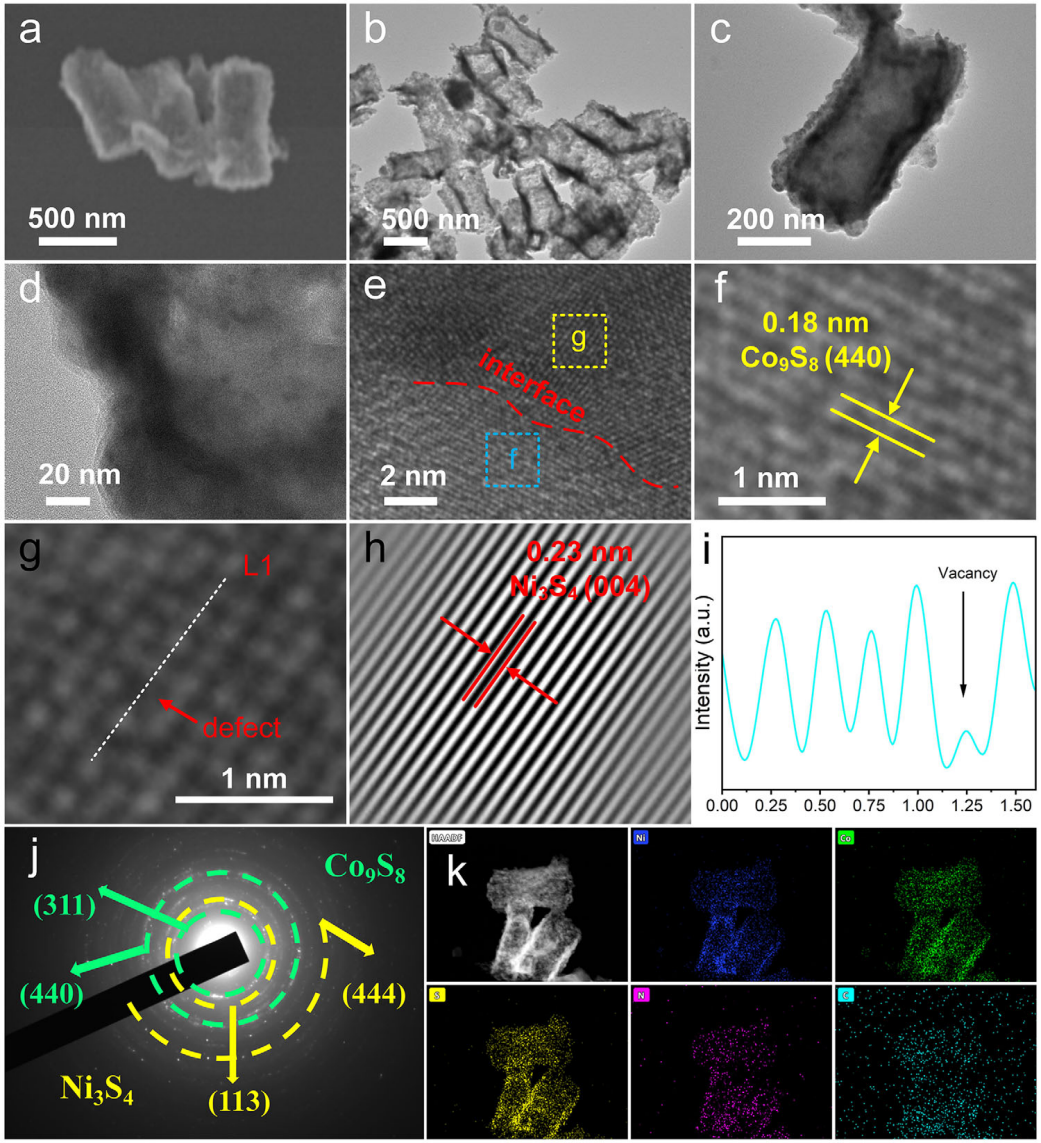
Characterizations of the as‐synthesized Ni_3_S_4_/Co_9_S_8_@C catalyst. a) SEM, b–d) TEM, e–g) HRTEM images, h) corresponding inverse‐FFT image of Ni_3_S_4_ domain; i) image intensity line profiles taken along the lines in Figure 4g; j) SAED pattern, k) element mapping.

To get more details of the morphological features and internal architecture of the as‐obtained Ni_3_S_4_/Co_9_S_8_@NC and Co_9_S_8_@NC catalysts, a series of SEM and TEM characterizations were performed. As shown in the SEM image of **Figures** **4**a and S10 (Supporting Information), the Ni_3_S_4_/Co_9_S_8_@NC and Co_9_S_8_@NC catalysts retained the hexagonal‐prismatic morphology of the corresponding intermediates. However, the TEM image clearly revealed a well‐defined hollow interior of the Ni_3_S_4_/Co_9_S_8_@NC sample (Figure 4b). The hollow structure is favorable for enhancing electrolyte infiltration and promoting the efficient release of gas bubbles during the water splitting process. A representative TEM image of an individual nanoprism was shown in Figure 4c, which further confirms the hollow structure of the as‐obtained Ni_3_S_4_/Co_9_S_8_@NC catalyst. Particularly, the shell is composed of densely packed and interconnected small nanoparticles, which not only maximizes the surface‐to‐volume ratio but also facilitates charge and mass transport.^[^ ^44^ ^]^ The magnified TEM image in Figure 4d reveales that the thickness of the shell was ~50 nm. The specific surface area of the Ni_3_S_4_/Co_9_S_8_@NC catalyst is determined to be 45.86 m^2^ g^−1^ based on the Brunauer–Emmett–Teller (BET) analysis of nitrogen absorption/desorption isotherm, larger than that of 27.90 m^2^ g^−1^ for the Co_9_S_8_@NC sample (Figure S11, Supporting Information). The enlarged surface area is favorable for increasing the catalytic sites for HER and OER. Additionally, an amorphous carbon layer was observed on the surface of each particle derived from the in situ carbonization of PVP during annealing. This conclusion is supported by the Raman measurement. As shown in Figure S12 (Supporting Information), two obvious characteristic peaks at ~1347 cm^−1^ (D band) and ~1580 cm^−1^ (G band) confirm the presence of N‐doped carbon in the sample. The I_D_/I_G_ ratio was calculated to be 1.055, indicating a relatively high density of defects and structural disorder. Interestingly, this N‐doped carbon layer is conducive to improving the conductivity of the electrode and buffering mechanical stress during long‐term operation.^[^ ^45^ ^]^ The existence of Ni_3_S_4_/Co_9_S_8_ heterojunction was directly visualized in the high‐resolution TEM (HRTEM) image of Figure 4e. The interplanar spacings of 0.23 and 0.18 nm were assigned to the (440) plane of Co_9_S_8_ (Figure 4f) and the (004) plane of Ni_3_S_4_ (Figure 4g,h), respectively. Notably, the line intensity profile along region L1 presented in Figure 4i suggests the presence of undercoordinated surface atom, which serves as active centers for H^*^ adsorption and intermediate stabilization, thereby contributing to improved HER catalytic activity.^[^ ^16^ ^]^ The selected‐area electron diffraction (SAED) pattern displayed concentric rings corresponding to both Ni_3_S_4_ and Co_9_S_8_ phase (Figure 4j), respectively, consistent with the XRD results. Moreover, the elemental mapping obtained via high‐angle annular dark‐field scanning transmission electron microscopy (HAADF‐STEM) revealed the uniform distribution of Ni, Co, S, N, and C in the product (Figure 4k). The energy‐dispersive X‐ray spectroscopy (EDX) analysis yielded an approximate atomic ratio of Ni:Co:S:N:C ≈ 17.37:17.13:31.07:10.81:23.62, corresponding to 23.62 wt.% of carbon in the catalyst. These results confirm both the existence of the N‐doped carbon and its homogeneous dispersion in Ni_3_S_4_/Co_9_S_8_@NC (Figure S13, Supporting Information).

### 2.3 Electrocatalytic HER Performance

**Figure 5:**
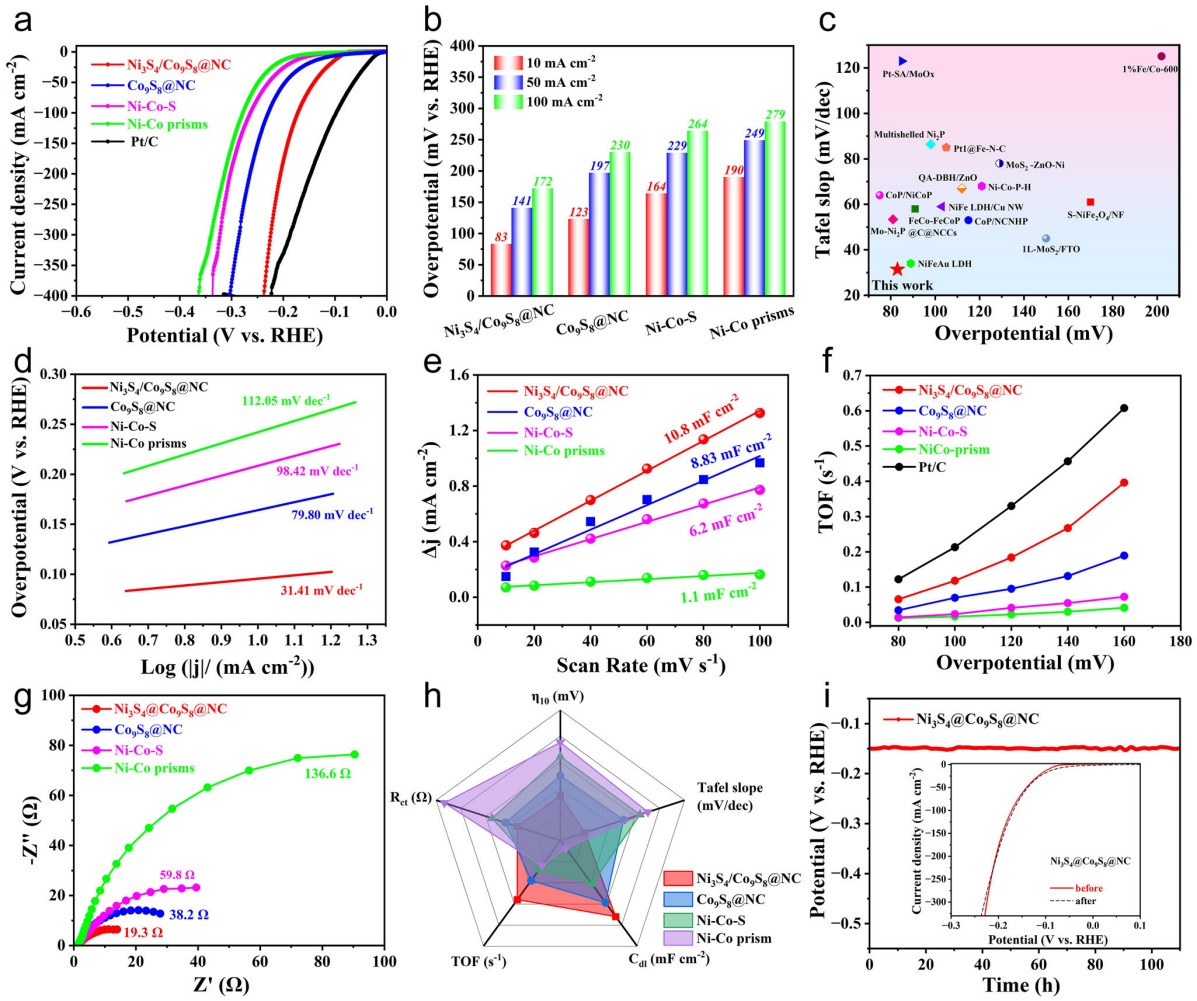
a) IR‐corrected LSV curves of the Ni_3_S_4_/Co_9_S_8_@NC, Co_9_S_8_@NC, Ni‐Co‐S, and NiCo‐prism catalysts with 20% Pt/C for comparison. b) Summary of the overpotentials at the current density of 10, 50, and 100 mA cm^−2^. c) Comparison of the overpotentials at 10 mA cm^−2^ and the corresponding Tafel slopes with other state‐of‐the‐art electrocatalysts. The Tafel plots (d), fitted C_dl_ (e), TOFs (f), and EIS spectrum (g) of the above electrodes. h) Systematic comparison of HER activities between Ni_3_S_4_/Co_9_S_8_@NC and reference catalysts. i) The chronopotentiometry curves of the Ni_3_S_4_/Co_9_S_8_@NC catalysts at 10 mA cm^−2^. The insert showed the LSV curves of Ni_3_S_4_/Co_9_S_8_@NC before and after 1000 cycles.

The HER activity of the Ni_3_S_4_/Co_9_S_8_@NC catalyst was evaluated in a standard three‐electrode system using 1 m KOH electrolyte. For comparison, the samples of Co_9_S_8_@C, Ni‐Co‐S, Ni‐Co nanoprism and Pt/C were tested under identical conditions. The linear sweep voltammetry (LSV) curves acquired at a scan rate of 5 mV s^−1^ with 90%‐iR compensation were displayed in **Figure** **5**a. It can be seen that the Ni_3_S_4_/Co_9_S_8_@NC catalyst displayed excellent HER catalytic activity, requiring a η_10_ of only 83 mV, outperforming the Co_9_S_8_@NC (123 mV), Ni‐Co‐S (164 mV), and Ni‐Co nanoprisms (190 mV). The reduced η_10_ for Ni_3_S_4_/Co_9_S_8_@NC strongly implies the synergistic contributions from defect engineering, shape control, and interfacial electronic structure regulation, which maximizes the number of active sites and facilitates the rapid charge and mass transfer. A comparative histogram of overpotentials to reach the current densities of 10, 50, and 100 mA cm^−2^ was summarized in Figure 5b. The Ni_3_S_4_/Co_9_S_8_@NC catalyst maintained lower overpotentials compared to other samples, demonstrating its excellent HER electrocatalytic activity. Although the η_10_ of Ni_3_S_4_/Co_9_S_8_@NC was higher than Pt/C (27.8 mV), it was smaller than or comparable to many previously reported Ni and Co‐based sulfides (Figure 5c; Table S1, Supporting Information), highlighting its promising application for alkaline water splitting. The Tafel slopes were then extracted from LSV plots to gain insights into HER kinetics and rate‐determining steps. As shown in Figure 5d, the Ni_3_S_4_/Co_9_S_8_@NC exhibited the lowest Tafel slope of 31.41 mV dec^−1^ compared to 79.8 mV dec^−1^ for Co_9_S_8_@NC, 98.42 mV dec^−1^ for Ni‐Co‐S, and 112.05 mV dec^−1^ for Ni‐Co nanoprisms, indicating its fastest HER kinetics. Additionally, the Tafel slopes were within the range of 30–120 mV dec^−1^, suggest that the charge transfer‐induced water dissociation on the surface of catalysts is the rate‐determining step, following the Volmer–Tafel mechanism.

To further validate the superior HER catalytic activity of the as‐fabricated Ni_3_S_4_/Co_9_S_8_@NC catalyst, the electrochemically active surface areas (ECSA) were estimated based on the calculation of double‐layer capacitance (C_dl_), which can be obtained from the cyclic voltammetry (CV) measurements performed in the non‐Faradaic potential region (Figure S14, Supporting Information). Given that ECSA is directly proportional to C_dl_, a larger C_dl_ indicates a higher density of accessible catalytic sites. As illustrated in Figure 5e, the Ni_3_S_4_/Co_9_S_8_@NC exhibited a C_dl_ value of 10.8 mF cm^−2^ with the scan rates ranging from 10 to 100 mV s^−1^ exceeding those of the Co_9_S_8_@NC (8.83 mF cm^−2^), Ni‐Co‐S (6.2 mF cm^−2^) and Ni‐Co prisms (1.1 mF cm^−2^), confirming that the hollow heterostructure and heterostruction effectively enhances surface exposure and increases the number of active sites available for HER. To quantitatively evaluate the intrinsic HER activity of the above catalysts, the turnover frequencies (TOFs) were calculated, as shown in Figure 5f. Across the overpotential range of 100–180 mV, the Ni_3_S_4_/Co_9_S_8_@NC catalyst exhibited higher TOF values, confirming its superior intrinsic HER catalytic activity. Particularly, at an applied overpotential of 120 mV versus RHE, the Ni_3_S_4_/Co_9_S_8_@NC catalyst achieved a TOF of 0.18 s^−1^ much higher than those of Co_9_S_8_@NC (0.09 s^−1^), Ni‐Co‐S (0.04 s^−1^), and NiCo‐nanoprisms (0.02  s^−1^), demonstrating that the integration of defective Ni_3_S_4_ and Co_9_S_8_ greatly enhanced the HER kinetics. Moreover, electrochemical impedance spectroscopy (EIS) was recorded to investigate the charge transfer kinetics at the electrode–electrolyte interface. The resulting Nyquist plots in Figure 5g exhibited the smallest semicircle diameter of Ni_3_S_4_/Co_9_S_8_@NC, corresponding to a low charge transfer resistance (R_ct_) of 19.3 Ω. This value is much lower than those of Co_9_S_8_@NC (38.2 Ω), Ni‐Co‐S (59.8 Ω), and Ni‐Co prisms (136.6 Ω), indicating its accelerated interfacial electron transfer kinetics. A systematic comparison of η_10_, Tafel slopes, C_dl_, TOFs, and R_ct_ is presented in Figure 5h, revealing that the Ni_3_S_4_/Co_9_S_8_@NC catalyst exhibited markedly superior performance over the control samples, underscoring the decisive role of hydrogen spillover in accelerating HER kinetics.

In addition to electrocatalytic activity, the long‐term stability is another key parameter for assessing the practical applicability of a HER catalyst. To evaluate the durability of Ni_3_S_4_/Co_9_S_8_@NC catalyst, the chronopotentiometric measurement was conducted at a constant current density of 10 mA cm^−2^ for 100 h. As shown in Figure 5i, the potential nearly unchanged, demonstrating its excellent long‐term stability. Additionally, the LSV curves recorded before and after 1000 CV cycles showed negligible differences, further confirming its robust durability (insert of Figure 5i). To assess industrial applicability, the HER stability of the Ni_3_S_4_/Co_9_S_8_@NC was then tested at a high current density of 1000 mA cm^−2^ for 35 h. The potential was largely maintained with negligible decay, indicating its strong structural integrity and remarkable tolerance under demanding operating conditions (Figure S15, supporting information).

### 2.4 Experimental and Theoretical Confirmation of Hydrogen Spillover

**Figure 6:**
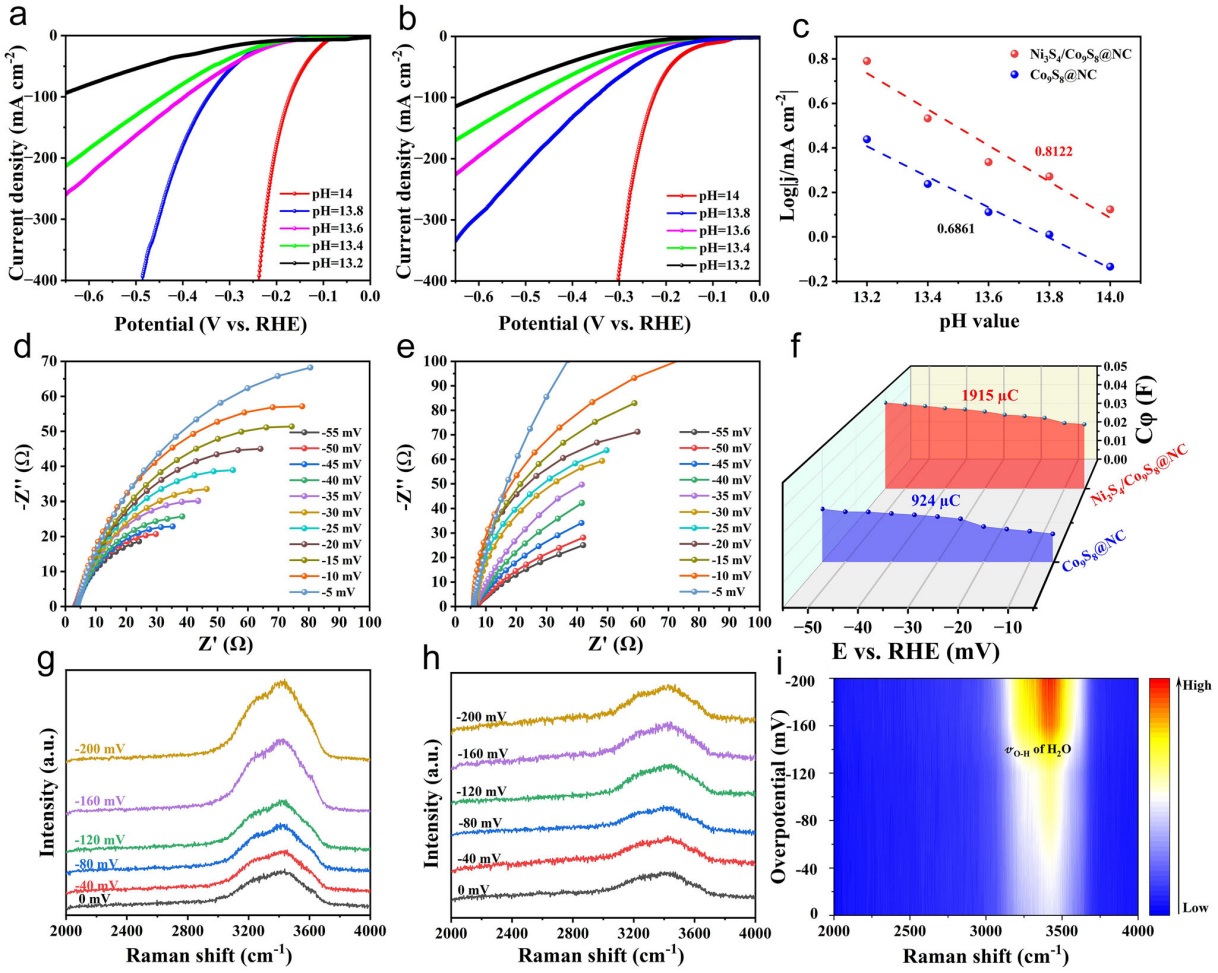
HER performance of Ni_3_S_4_/Co_9_S_8_@NC (a) and Co_9_S_8_@NC (b) in 1.0 m KOH with various pH. c) The linear plots of log|j@−50 mV versus RHE| versus pH. EIS spectra of Ni_3_S_4_/Co_9_S_8_@NC (d) and Co_9_S_8_@NC (e) at various potentials. f) Plot of C_1_ versus η for the two catalysts. In‐situ Raman spectra of Ni_3_S_4_/Co_9_S_8_@NC (g) and Co_9_S_8_@NC (h) with different operated potentials (vs. RHE), and the 2D contour image (i,) of Ni_3_S_4_/Co_9_S_8_@NC.

To verify the hydrogen spillover mechanism in the Ni_3_S_4_/Co_9_S_8_@NC catalyst, a series of electrochemical measurements were conducted to clarify the correlations between interfacial electron redistribution and hydrogen spillover behaviors. According to previous studies, the hydrogen spillover mechanism for HER typically occurs in electrocatalysts with a Tafel slope close to or below 30 mV dec^−1^.^[^ ^23^, ^46^ ^]^ As shown in Figure 5a, the Ni_3_S_4_/Co_9_S_8_@NC catalyst exhibited a Tafel slope of 31.41 mV dec^−1^ suggesting the possible hydrogen spillover mechanism. To further support this hypothesis, the HER catalytic activity was evaluated in KOH with different pH values. As illustrated in **Figure** **6**a,b, the HER performance of the Ni_3_S_4_/Co_9_S_8_@NC catalyst improved with the increase of pH value. The reaction order with respect to pH, derived from the linear relationship between log|j@50 mV versus RHE| and pH, was found to be 0.812 for Ni_3_S_4_/Co_9_S_8_@NC and 0.686 for Co_9_S_8_@NC, respectively (Figure 6c). The higher reaction order observed for Ni_3_S_4_/Co_9_S_8_@NC indicated the enhanced hydrogen spillover effect by the heterointerface, which facilitates the migration of H^*^ intermediates between distinct catalytic sites.^[^ ^47^ ^]^ This interfacial synergy not only promotes water dissociation but also optimizes subsequent hydrogen evolution steps, collectively leading to an increased pH‐dependence of the overall HER rate.

In situ EIS was then performed to inspect the charge transfer dynamics at the electrolyte‐electrocatalyst interface. As shown in Figure 6d,e, the diameter of the Nyquist semicircles decreased systematically with the increase of overpotentials. According to the equivalent circuit, The R_ct_ and CPE represent the charge transfer resistance and constant phase element, respectively (Figure S16, Supporting Information). The Ni_3_S_4_/Co_9_S_8_@NC exhibited a lower R_ct_ than Co_9_S_8_@NC (Tables S2 and S3, Supporting Information), indicating much faster interfacial electron transfer kinetics.^[^ ^48^ ^]^ Additionally, the C_φ_ and R_2_ represent the pseudocapacitance and resistance associated with hydrogen adsorption, respectively. The hydrogen adsorption charge (Q_H_), calculated by integrating C_φ_ with respect to the overpotential, quantitatively reflects the amount of hydrogen adsorbed on the catalyst surface. As shown in Figure 6f, the Q_H_ value for Ni_3_S_4_/Co_9_S_8_@NC was nearly two times higher than that of Co_9_S_8_@NC, demonstrating its much enhanced hydrogen adsorption capacity. Moreover, the smaller R_2_ for Ni_3_S_4_/Co_9_S_8_@NC suggests the lower hydrogen adsorption resistance and faster adsorption kinetics, which facilitates the migration of H^*^ species from Co_9_S_8_ to Ni_3_S_4_, thereby increasing the amount of active hydrogen on Ni_3_S_4_.

To further confirm the interfacial hydrogen spillover behaviors, electrochemical in‐situ Raman measurements were carried out. The Raman spectra were recorded at incremental potentials from 0 to −200 mV versus RHE. As shown in Figure 6g,h, a broad Raman band centered at 3420 cm^−1^ was detected for the Ni_3_S_4_/Co_9_S_8_@NC and Co_9_S_8_@NC samples, which was typically assigned to interfacial water molecules adsorbed on their surface. Interestingly, the Raman intensity gradually intensified as the potential shifted negatively. Particularly, the Ni_3_S_4_/Co_9_S_8_@NC catalyst exhibited a sharper enhancement of intensity beginning at –160 mV, in contrast to the more gradual evolution observed for Co_9_S_8_@NC, suggesting a more favorable environment for water adsorption and activation at the heterostructured interface (Figures 6i; S17, Supporting information).^[^ ^49^ ^]^ Given the electronic and structural differences between the Co_9_S_8_ and Ni_3_S_4_ domains, it is reasonable to infer that initial H_2_O molecules dissociation occurs preferentially at the Co_9_S_8_ sites, and the resulting H^*^ intermediates subsequently migrate to Ni_3_S_4_. This directional transfer is consistent with a hydrogen spillover mechanism operating across the interface. Considered alongside the higher HER reaction order with respect to pH value, the in‐situ Raman results offer complementary evidence for the enhanced interfacial coupling in the Ni_3_S_4_/Co_9_S_8_@NC catalyst.

**Figure 7:**
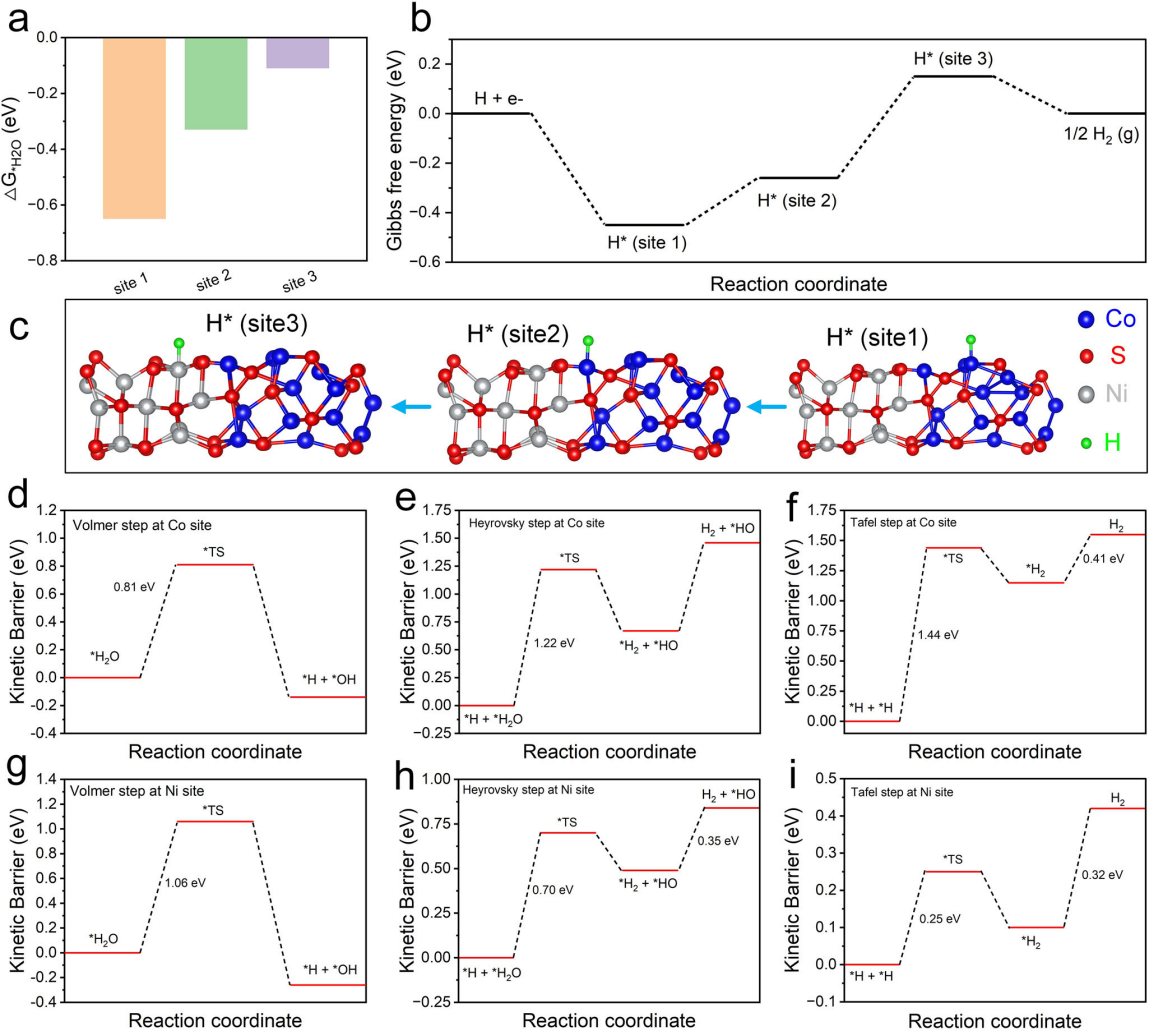
DFT calculations of the hydrogen spillover mechanism. a) The adsorption free energy of H_2_O at different reaction sites. The calculated free energy diagram (b) and DFT‐optimized model (c) of hydrogen evolution on different sites. The alkaline HER process of the Co site (d–f), and Ni site (g–i).

DFT calculations were also conducted to substantiate the proposed hydrogen spillover mechanism, revealing how interfacial electronic structure modulation and reduced migration barriers account for the improved HER performance. In the alkaline medium, the HER initiates with the adsorption of H_2_O molecules. As shown in **Figure** **7**a, the calculated adsorption energies (E_ads_) of H_2_O on the Co_9_S_8_/V_s_‐Ni_3_S_4_ heterostructure are −0.65 eV for the Co site (site 1), −0.33 eV for the bridge Co site (site 2), and −0.11 eV for the Ni site (site 3), respectively. Notably, the lower E_ads_ values at Co sites compared to the Ni site suggest its stronger H_2_O adsorption due to the high‐valence Co centers which serve as effective active sites.^[^ ^50^ ^]^ Subsequently, the energy barriers for H_2_O dissociation via the Volmer‐step were evaluated. The calculated activation energies are 0.81 eV at the Co site (Figure 7d) and 1.06 eV at the Ni site (Figure 7g), respectively, indicating that H_2_O molecules are more readily dissociated at the Co sites. The resulting ^*^H and ^*^OH intermediates are stabilized via their adsorption on adjacent electron‐deficient Co and electron‐rich S sites, facilitating the initial step of HER.^[^ ^51^ ^]^ The thermodynamics of the hydrogen intermediate transfer were further assessed. At the site 1 and site 2, ∆G_H*_ < 0, indicating strong H^*^ adsorption energy of Co site, whereas at the site 3, ∆G_H*_ > 0, suggesting weak H^*^ adsorption favorable for H_2_ desorption (Figure 7b). These results indicate that H^*^ generated at Co sites can transfer to adjacent Ni_3_S_4_ domains, enabling a directional spillover process (Figure 7c). Notably, the ΔG_H*_ at the Ni site was calculated to be 0.11 eV, indicating optimal hydrogen binding strength and superior HER performance. The subsequent H_2_ formation steps were further analyzed via the Heyrovsky (H_2_O + H^*^ + e^−^ → H_2_ + OH^−^) and Tafel (^*^H + ^*^H → H_2_) pathways. As shown in Figure 7e,f, the kinetic barriers for H_2_ formation at Co sites is relatively high, suggesting that Co_9_S_8_ is more suitable for the initial water dissociation rather than for H_2_ formation and release. In contrast, the Ni site (site 3) exhibits much lower energy barriers for both Heyrovsky (Figure 7h) and Tafel steps (Figure 7i). These DFT calculation results demonstrated a bifunctional mechanism enabled by the synergistic Co_9_S_8_ and V_s_‐Ni_3_S_4_ interface, where Co sites catalyze water dissociation and H^*^ generation, and sulfur vacancy‐rich Ni_3_S_4_ sites facilitate hydrogen spillover and promote the subsequent H_2_ formation. The enhanced spatial charge redistribution across the heterojunction, leads to a substantial reduction in both thermodynamic and kinetic barriers throughout the HER pathway, thereby delivering significantly improved catalytic performance.

### 2.5 Electrocatalytic OER Performance

**Figure 8:**
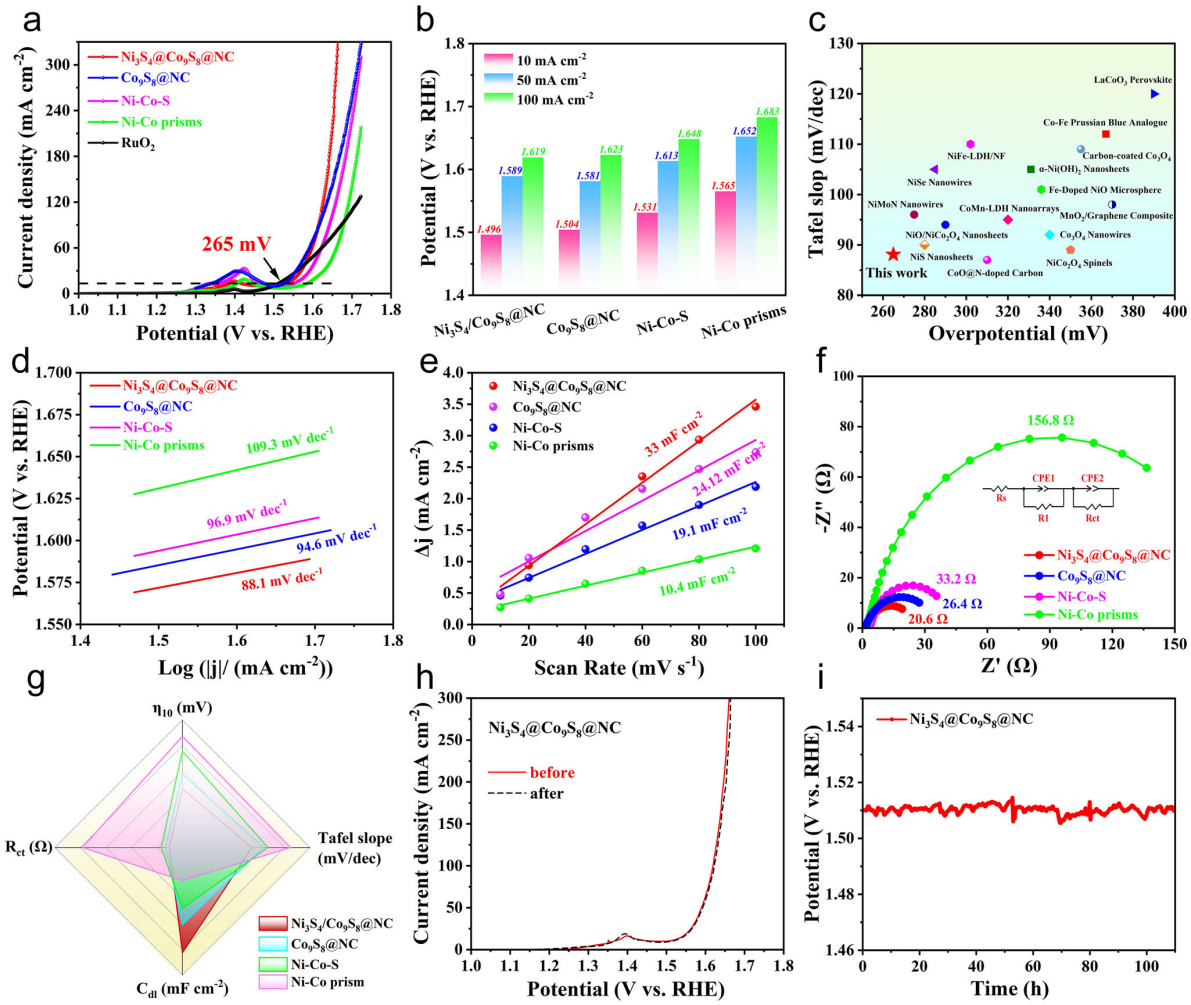
OER performance of the as‐synthesized catalysts. a) LSV curves of the as‐synthesized Ni_3_S_4_/Co_9_S_8_@NC, Co_9_S_8_@NC, Ni‐Co‐S and NiCo‐prisms samples; b) The summary of the overpotentials at 10, 50, and 100 mA cm^−2^. c) Comparison of the η_10_ and the corresponding Tafel slopes with other OER electrocatalysts. d) Tafel slope; e) C_dl_, f) EIS spectra of different catalysts. g) Systematic comparison of OER performance between Ni_3_S_4_/Co_9_S_8_@NC and reference catalysts. h) LSV plots before and after 1000 cycles. i) The long‐term stability of the Ni_3_S_4_@Co_9_S_8_@NC catalyst.

To assess the bifunctional catalytic activity of the Ni_3_S_4_/Co_9_S_8_@NC catalyst, OER performance was also evaluated in 1.0 m KOH using a standard three‐electrode system. The IR‐corrected LSV curves exhibited an oxidation peak at ~1.4 V versus RHE (**Figure** **8**a), attributed to the oxidation of Ni^2+^ species. At a benchmark current density of 10 mA cm^−2^, the Ni_3_S_4_/Co_9_S_8_@NC achieves a low overpotential of 265 mV, outperforming the Co_9_S_8_@NC (302 mV), Ni‐Co‐S (339 mV), and Ni‐Co prisms (368 mV). The potential comparison across varying current densities demonstrates the excellent adaptability of Ni_3_S_4_/Co_9_S_8_@NC (Figure 8b). As illustrated in Figure 8c and Tables S4 (Supporting Information), the Ni_3_S_4_/Co_9_S_8_@NC also delivered superior OER activity compared to the other Ni/Co‐based compounds. The Tafel slopes derived from the LSV plots showed that Ni_3_S_4_/Co_9_S_8_@NC achieved a value of 88.1 mV dec^−1^, smaller than the Co_9_S_8_@NC (94.6 mV dec^−1^), Ni‐Co‐S (96.9 mV dec^−1^) and Ni‐Co prisms (109.3 mV dec^−1^) (Figure 8d), uncovering the critical roles of hollow structure design, component control and sulfur vacancy engineering in accelerating OER kinetics. As shown in Figures 8e and S18 (Supporting Information), the Ni_3_S_4_/Co_9_S_8_@NC catalyst exhibited a C_dl_ of 33.0 mF cm^−2^, substantially higher than those of the Co_9_S_8_@NC (24.12 mF cm^−2^), Ni‐Co‐S (19.1 mF cm^−2^), and Ni‐Co nanoprisms (10.4 mF cm^−2^). The higher C_dl_ value indicated that Ni_3_S_4_/Co_9_S_8_@NC possessed an increased active surface area, which contributed to its superior OER performance. The EIS Nyquist plots in Figure 8f showed that the Ni_3_S_4_/Co_9_S_8_@NC exhibited the smallest R_ct_ of 20.6 Ω, indicating its rapid electron transfer at the catalyst‐electrolyte interface.

To evaluate the OER durability of the Ni_3_S_4_/Co_9_S_8_@NC catalyst, LSV curves were measured before and after 1000 CV cycles, and no significant variation was observed, suggesting that its intrinsic OER activity was well preserved (Figure 8h). Additionally, the chronopotentiometric measurement at 10 mA cm^−2^ showed that the potential remained essentially unchanged over 100 h, confirming its long‐term stability (Figure 8i). Moreover, even under a high current density of 1000 mA cm^−2^, the catalyst maintained stable for 30 h with minimal degradation (Figure S19, Supporting Information). These results demonstrate that the catalyst possesses high structural robustness and long‐term electrochemical stability. To gain deeper insights into its surface dynamic reconstruction during the OER process, the morphological and structural evolution was then examined. As shown in Figure S20 (Supporting Information), the SEM image showed no obvious changes after the OER process. However, the TEM images showed that the surface of the nanocubes had an emerging thin layer. Additionally,   the interplanar spacings of 0.21 and 0.23 nm can be indexed into the (210) plane of NiOOH and the (111) plane of CoOOH, respectively. These results indicate that after the OER activation process, the surface of Co_9_S_8_/Ni_3_S_4_@NC catalyst was partially oxidized to OER‐active (oxy)hydroxides.

To further elucidate the surface reconstruction mechanism, in situ Raman spectra of were collected at open‐circuit potential (OCP) and at potentials ranging from 1.2 to 1.6 V (Figure S21, Supporting Information). For Co_9_S_8_/Ni_3_S_4_@NC, potential‐dependent peaks emerged from 1.3 V and gradually increased in intensity with rising potential. The strong band at ~451 cm^−1^ can be attributed to the E_g_ bending vibration of Ni/Co─O, while the feature near 575 cm^−1^ corresponds to the A_1g_ stretching vibration of Ni/Co─O in oxyhydroxides, confirming the formation of NiOOH/CoOOH species.^[^ ^10^, ^52^ ^]^ In contrast, the Co_9_S_8_@NC sample exhibited weaker intensity in the same potential range, suggesting a lower degree of surface reconstruction. Additionally, a peak ≈746 cm^−1^ gradually diminished above 1.5 V, implying that the initially ordered oxide lattice was further oxidized into a more disordered structure under strong anodic polarization. These Raman observations are well supported by the post‐OER XPS results (Figure S22, Supporting Information). In the Ni 2p spectrum, peaks at 877.2 and 856.8 eV can be assigned to Ni^3+^ 2p3/2 and the satellite feature, respectively, indicating partial oxidation of Ni species.^[^ ^52^ ^]^ A similar trend is observed in the high‐resolution Co 2p spectrum. Furthermore, in the S 2p spectrum, the relative intensity of sulfate species increases after OER, suggesting oxidation of surface sulfur anions under high potentials. The strong O 1s peak after OER further supports the surface oxidation of the Co_9_S_8_/Ni_3_S_4_@NC catalyst.^[^ ^53^ ^]^ These results indicate that the Co_9_S_8_/Ni_3_S_4_@NC catalyst underwent surface reconstruction into oxide/oxyhydroxide species. At the same time, the conductive Co_9_S_8_/Ni_3_S_4_@NC framework serves as a robust scaffold. The strong interfacial coupling between the reconstructed surface and the underlying sulfide phase facilitates efficient charge transfer, thereby enhancing the OER activity and maintaining long‐term durability.

On the basis of the above electrochemical results and DFT calculations, the as‐designed Co_9_S_8_/Ni_3_S_4_@NC catalyst exhibits outstanding bifunctional activity for water splitting, which can be attributed to its well‐designed structure and electronic configuration. First, the hollow heterostructure provides large surface area, thus providing numerous active sites for HER and OER. Second, the sulfur vacancy engineering regulates the interfacial electronic structure by reducing the work function difference, weakening the built‐in electric field, thereby trigering hydrogen spillover and accelerating HER kinetics. Thirdly, the conductive sulfide framework ensures rapid electron transport and provides a robust scaffold to stabilize dynamically formed oxide/oxyhydroxide layers during OER, leading to enhanced durability. Finally, the carbon coating improves conductivity, prevents particle agglomeration, and protects the active sites from structural degradation, thereby ensuring long‐term stability of the Co_9_S_8_/Ni_3_S_4_@NC catalyst.

## 3 Conclusion

In summary, with DFT calculations and experimental characterizations, the mechanism how interfacial electronic modulation governs the hydrogen spillover behavior in Ni_3_S_4_/Co_9_S_8_@NC electrocatalysts was uncovered. It revealed that the large ΔΦ between Ni_3_S_4_ and Co_9_S_8_ induces excessive interfacial electron accumulation, leading to strong H^*^ adsorption at the heterojunction interface and impeding its migration. The introduction of sulfur vacancy into Ni_3_S_4_ effectively reduced the ΔΦ and diluted the interfacial charge density, thereby redistributing electrons away from the interface. This electronic regulation weakens H^*^ binding at the interface while enhancing H^*^ activation on the Ni_3_S_4_ domain, ultimately lowering both the thermodynamic and kinetic barriers for H^*^ transfer. These results were experimentally corroborated by the excellent HER activity of the Ni_3_S_4_/Co_9_S_8_@NC catalyst with a low overpotential of 83 mV at 10 mA cm^−2^ which exhibited the smallest ΔΦ and the most favorable charge‐transfer dynamics. This work highlights the important role of vacancy‐mediated interfacial charge redistribution in lowering the energy barrier for hydrogen spillover, offering insights into the design of efficient, low‐cost electrocatalysts based on defect‐engineered heterojunctions.

## Conflict of Interest

The authors declare no conflict of interest.

## Supporting information



Supporting Information

## Data Availability

The data that support the findings of this study are available from the corresponding author upon reasonable request.
