# Supplementary material for: Sulfur Vacancy‐Engineered Co9S8‐Ni3S4 Heterostructure as a Hydrogen Spillover Catalyst for Efficient Alkaline Water Splitting
Source: Adv Sci (Weinh). 2025 Oct 6;12(48):e13610. doi: 10.1002/advs.202513610 (PMC12752608; doi:10.1002/advs.202513610)
Supplement: Supplementary file 1 — Supporting Information [file ADVS-12-e13610-s001.docx]

Supporting Information

Sulfur vacancy-engineered Co_9_S_8_-Ni_3_S_4_ heterostructure as a hydrogen spillover catalyst for efficient alkaline water splitting

Shoushuang Huang*, Tianyu Jin, Jie Zhang, Yong Jiang*, Jiwen Hu, Hejingying Niu*, Amene Naseri, Kajsa Uvdal, Zhangjun Hu* and Jiujun Zhang

Dr. S. Huang, T. Jin, J. Zhang, Dr. H. Niu, Dr. A. Naseri, Prof. Y. Jiang

School of Environmental and Chemical Engineering, Shanghai University/Shanghai Key Laboratory of Atomic Control and Application of Inorganic 2D Supermaterials, Shanghai 200444, China

E-mail: sshuang@shu.edu.cn; niuhjy@shu.edu.cn; jiangyong@shu.edu.cn;

Dr. J. Hu, Dr. Z. Hu, Prof. K. Uvdal

Division of Molecular Surface Physics & Nanoscience, Department of Physics, Chemistry and Biology, Linköping University, Linköping 58183, Sweden;

E-mail: zhangjun.hu@liu.se

Dr. A. Naseri

Agricultural Biotechnology Research Institute of Iran (ABRII), Agricultural Research, Education, and Extension Organization (AREEO), Karaj, 3135933151, Iran

Prof. J. Zhang

College of Sciences/Institute for Sustainable Energy, Shanghai University, Shanghai 200444, China

1. **Experiment**

**1.1 Synthesis of Ni-Co nanoprisms**

The Ni-Co nanoprisms were synthesized *via* a modified polyol reduction method according to our previous report (*Huang. et al., RSC Adv., 2018, 8(11), 5992-6000; Chem. Eng. J., 2021, 127630*). Briefly, 1.28 g of Ni(CH_3_COO)_2_·4H_2_O and 1.28 g Co(CH_3_COO)_2_·4H_2_O were dissolved in 200 mL of anhydrous ethanol under ambient conditions. The mixture was ultrasonicated for 30 min to ensure complete dissolution. Subsequently, 3.0 g of polyvinylpyrrolidone (PVP, MW ≈ 55,000) was added as a surfactant and capping agent with continuous magnetic stirring. The resulting solution was stirred for 30 min at room temperature and then transferred to a 200 mL round-bottom flask equipped with a vigreux condenser and refluxed at 85°C in an oil bath for 4 h. After cooling to room temperature, the product was collected by centrifugation, washed several times with ethanol to remove residual PVP and acetate ions, and finally dried at 60 °C in a vacuum oven for 12 h. For comparison, the Co nanoprisms were synthesized with the same

**1.2 Synthesis of Ni-Co-S intermediate**

Typically, 60 mg of the as-prepared Ni-Co nanoprisms was added into 60 mL of ethanol and magnetically stirred for 20 min to obtain a homogeneous mixture. Subsequently, 0.5 mL of ammonium sulfide solution (15 wt% (NH_4_)_2_S in H_2_O, Aladdin) was added dropwise to the mixture. After stirring for 30 min, the resulting product was isolated by centrifugation and sequentially washed with deionized water and ethanol to remove the residual ions and organics impurities, followed by vacuum drying at 60°C for 12 h.

**1.3 Synthesis of Ni_3_S_4_/Co_9_S_8_@NC**

For the synthesis of Ni_3_S_4_/Co_9_S_8_@NC, 30 mg of the Ni-Co-S power was loaded into an alumina combustion boat, covered with a quartz slide to minimize vapor loss, and annealed in a tube furnace under the protection of N_2_ with a flow of 50 sccm. The furnace was heated from room temperature to 350°C with a heating rate of 2°C min⁻^1^ and maintained at this temperature for 2 h. After that, the furnace was allowed to cool naturally to room temperature, and the catalyst of Ni_3_S_4_@Co_9_S_8_@NC was obtained.

**1.4 Material characterization**

The crystal structure and phase of the as-synthesized materials were characterized using powder X-ray diffraction (XRD, Rigaku D/MAX-2500, Cu-Kα radiation, λ = 1.5406 Å). The size and shape were analyzed via scanning electron microscopy (SEM, JEOL JSM-6700F) operated at 15 kV. The hollow and heterostructure was further elucidated by the transmission electron microscopy (TEM) and high-resolution TEM (HRTEM, JEOL JEM-2010F) at 200 kV acceleration voltage, coupled with selected-area electron diffraction (SAED) for crystallographic analysis. The surface elemental composition and chemical states were investigated by using X-ray photoelectron spectroscopy (XPS, Thermo Scientific ESCALAB 250Xi) with monochromatic Al-K_α_ excitation, calibrated against the C 1s peak at 284.8 eV. Raman EDX BET

**1.5 Electrocatalytic Measurements**

The electrocatalytic performance of the synthesized materials was evaluated using a CHI 660e electrochemical workstation within a standard three-electrode system. The catalyst-coated Ni foam (1 × 1 cm^2^, pre-cleaned via 3 M HCl etching and ethanol sonication) as the working electrode, a carbon rod counter electrode, and a Hg/HgO reference electrode. To prepare the working electrode, 6.0 mg of catalyst was dispersed in a mixture of 110 μL ethanol, 330 μL deionized water, and 40 μL Nafion solution (5 wt%) via 30 min ultrasonication treatment. Then, 120 μL of the resulting slurry was drop-cast onto the Ni foam substrate and air-dried at ambient temperature, yielding a uniform catalyst layer with a mass loading of 1.5 mg cm^–2^. For comparison, the standard RuO_2_ and Pt/C (20 wt%) electrodes were fabricated under identical conditions. All electrochemical tests were conducted in 1.0 M KOH electrolyte with 90% iR compensation, and the potentials were converted to the reversible hydrogen electrode (RHE) scale using the equation: *E*_RHE_ = *E*_Hg/HgO_ + 0.0592 × pH + 0.098V. Cyclic voltammetry (CV) was performed at scan rates ranging from 10 to 100 mV s⁻^1^ within a non-faradaic potential window to determine double-layer capacitances from the linear slope of current density versus scan rate. Electrochemical impedance spectroscopy (EIS) measurements spanned frequencies from 10^5^ Hz to 0.01 Hz with a 5 mV AC amplitude under open-circuit conditions.

**1.6 Computational Method**

The DFT calculations were performed using the Vienna ab initio Simulation Package with core-electron interactions described by the projector augmented wave (PAW) method and exchange-correlation effects approximated by the Perdew-Burke-Ernzerhof (PBE) generalized gradient approximation (GGA). A plane-wave basis set with a kinetic energy cutoff of 450 eV was employed, ensuring convergence of total energies within 10^–6^ eV. Structural relaxations proceeded until residual atomic forces fell below 0.02 eV Å^–1^. The Brillouin zone was sampled using a 2 × 2 × 1 Monkhorst-Pack k-point mesh, optimized for the slab models. The Ni_3_S_4_-Co_9_S_8_ heterostructure was constructed based on experimentally resolved lattice parameters from HRTEM analysis. A sulfur vacancy (V_S_) model was generated by removing one surface S atom from the pristine structure, followed by full atomic relaxation. All slab models incorporated a 15 Å vacuum layer along the z-axis to eliminate spurious periodic interactions. Notably, the model focused on the Co_9_S_8_/Ni_3_S_4_ heterostructure with and without sulfur vacancies, and did not explicitly include the N-doped carbon layer. This simplification was made to reduce computational complexity and to directly study the interfacial charge redistribution between the two phases. The Gibbs free energy (ΔG) profiles for catalytic intermediates were computed using the computational hydrogen electrode (CHE) model, where the chemical potential of (H⁺ + e⁻) pairs was referenced to H_2_ gas at standard conditions (pH = 0, 298 K, 1 bar). Charge density difference analysis and Bader charge partitioning quantified interfacial electron transfer between Ni_3_S_4_ and Co_9_S_8_ phases.


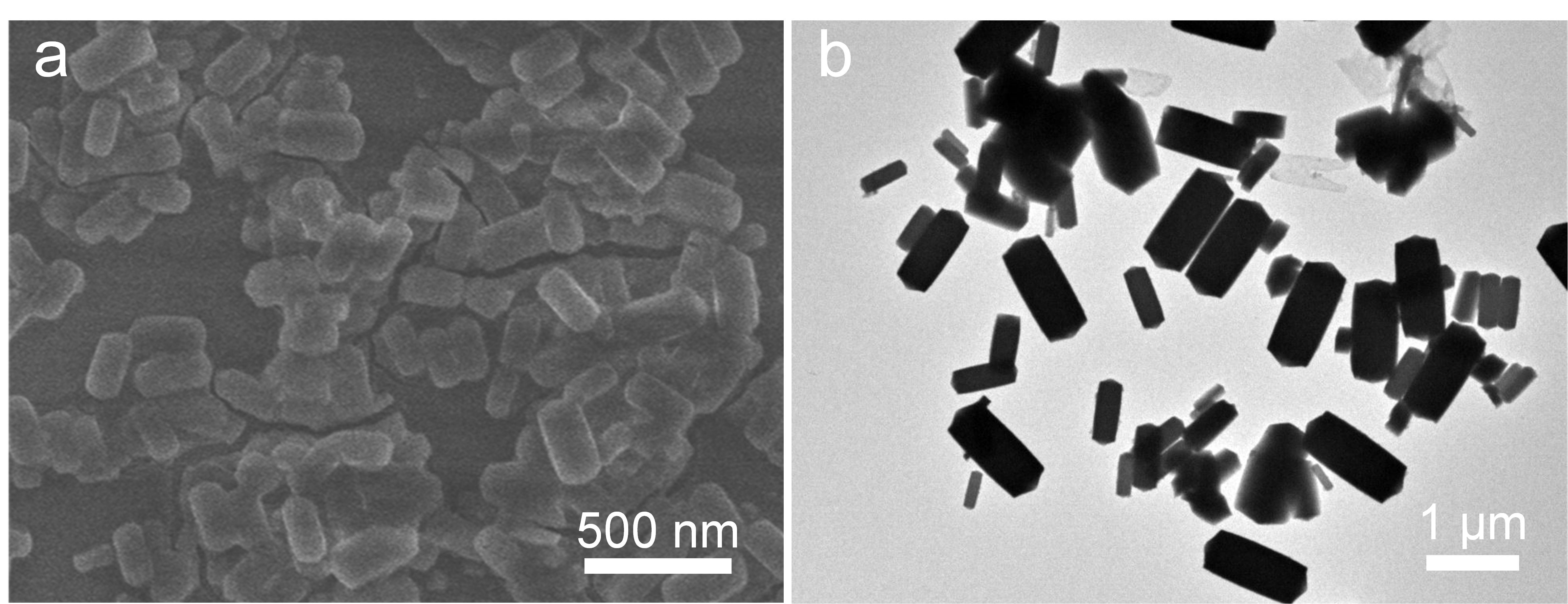


**Figure S1** The SEM (a) and TEM (b) images of the as-obtained Co-nanoprism.


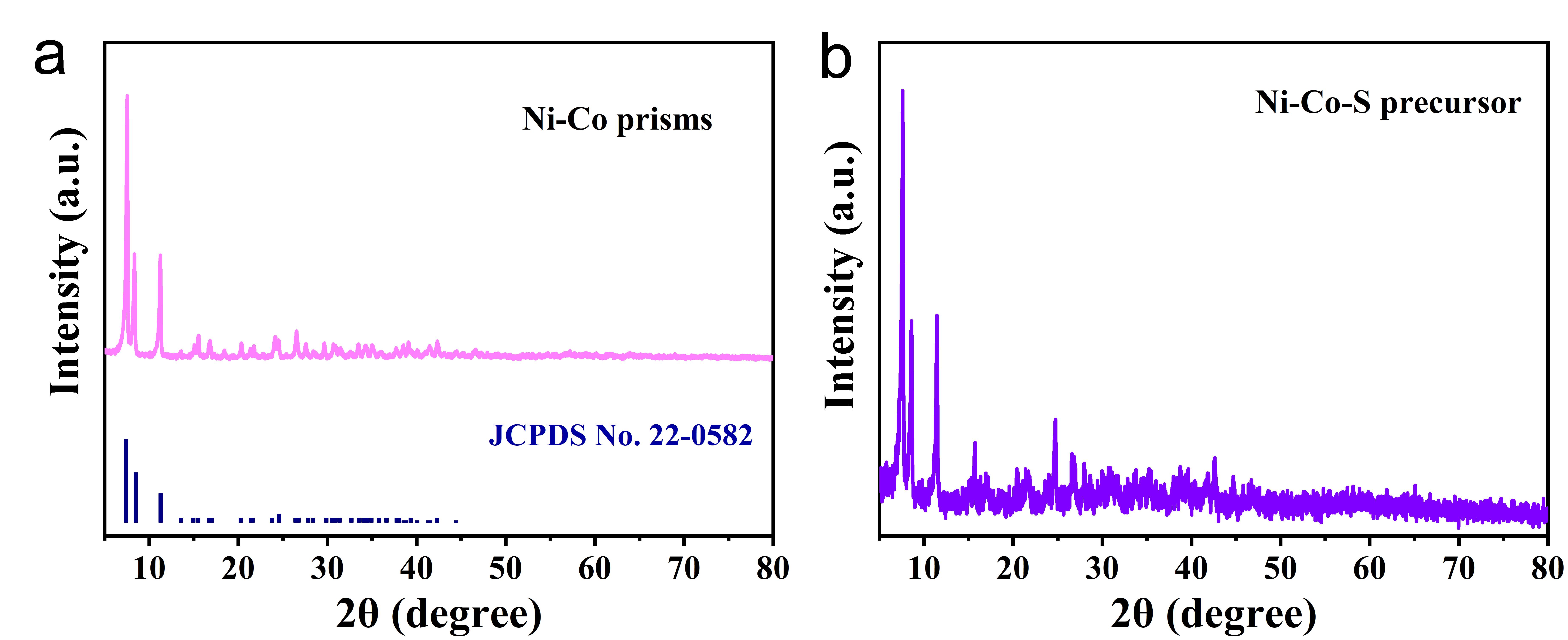


**Figure S2** XRD patterns of the Ni-Co nanoprisms before (a) and after (b) the (NH_4_)_2_S-mediated chemical reaction.


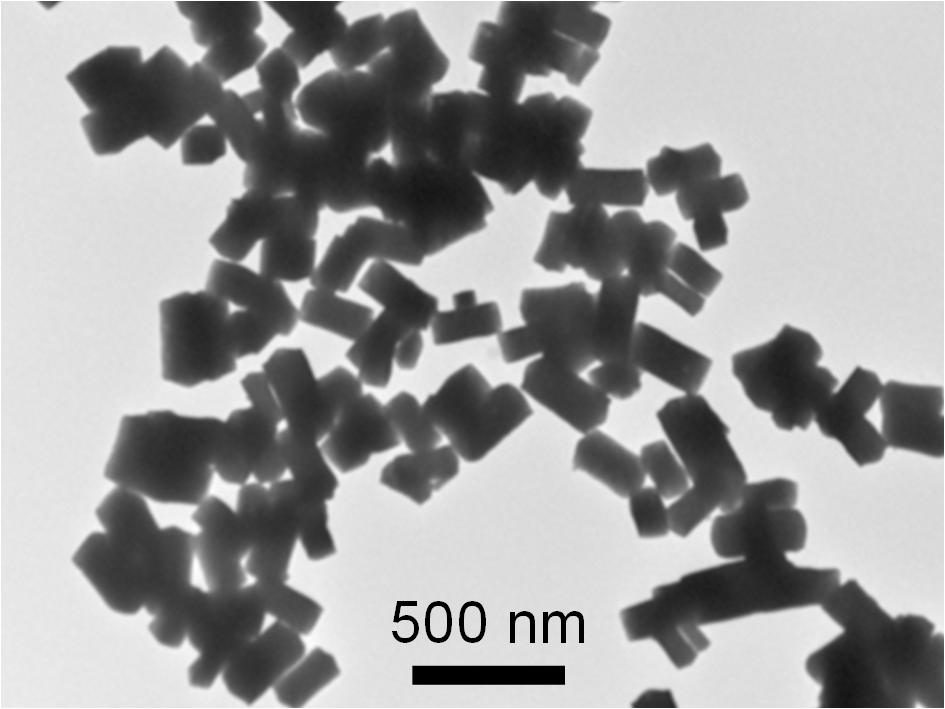


**Figure S3** TEM image of the as-obtained product with 0.1 mL (NH_4_)_2_S solution.


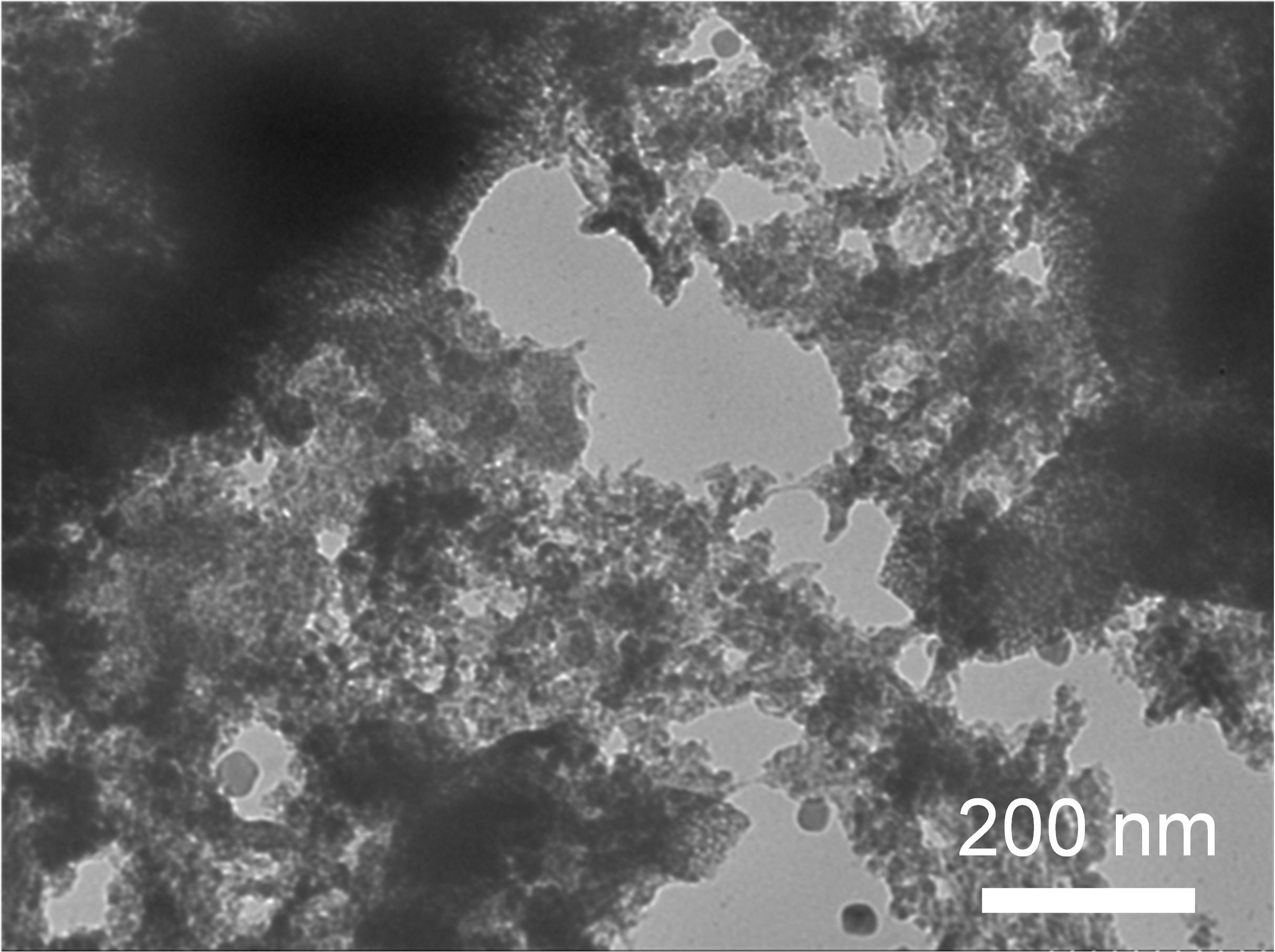


**Figure S4** TEM image of the as-obtained product with 1.0 mL (NH_4_)_2_S solution.


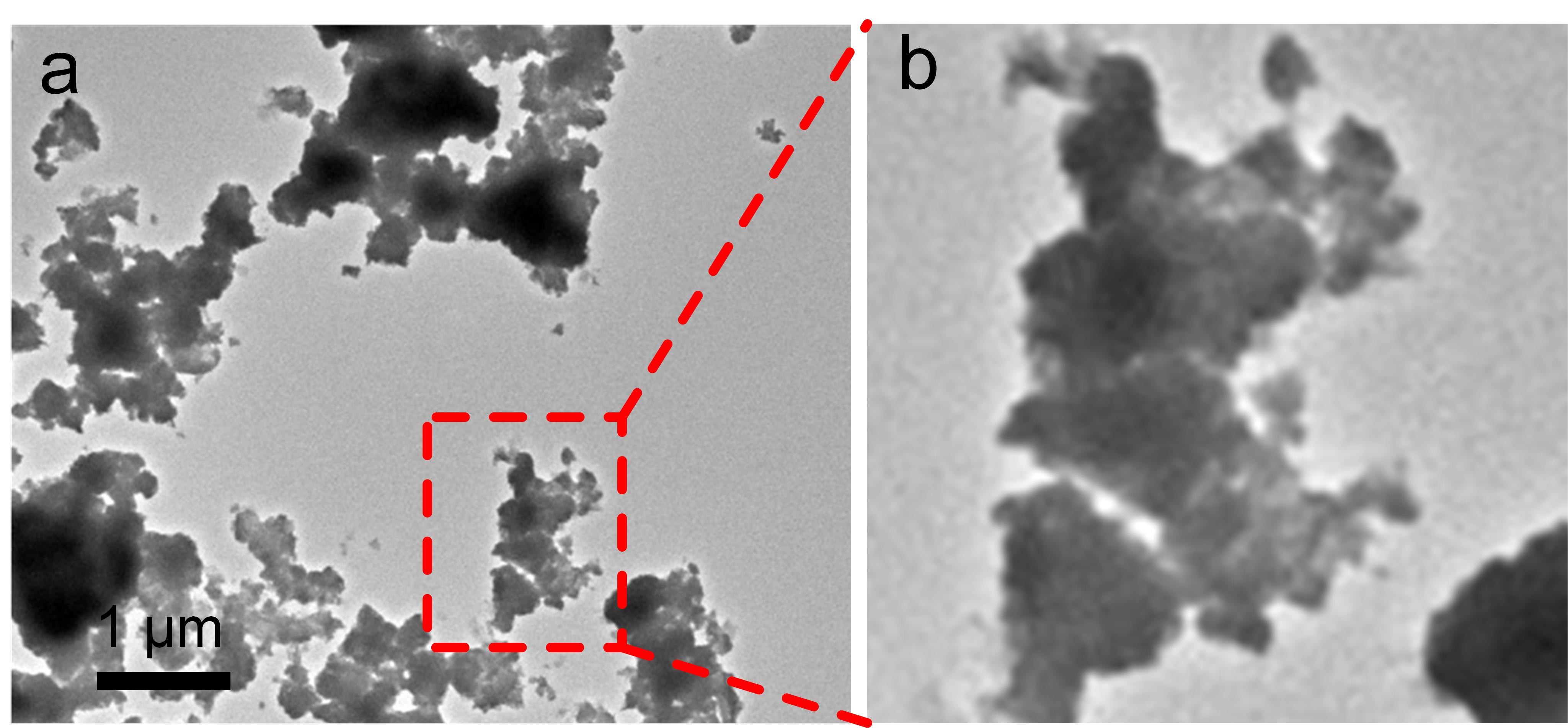


**Figure S5** TEM image of the product obtained at 50^o^C with 0.5 mL (NH_4_)_2_S solution.


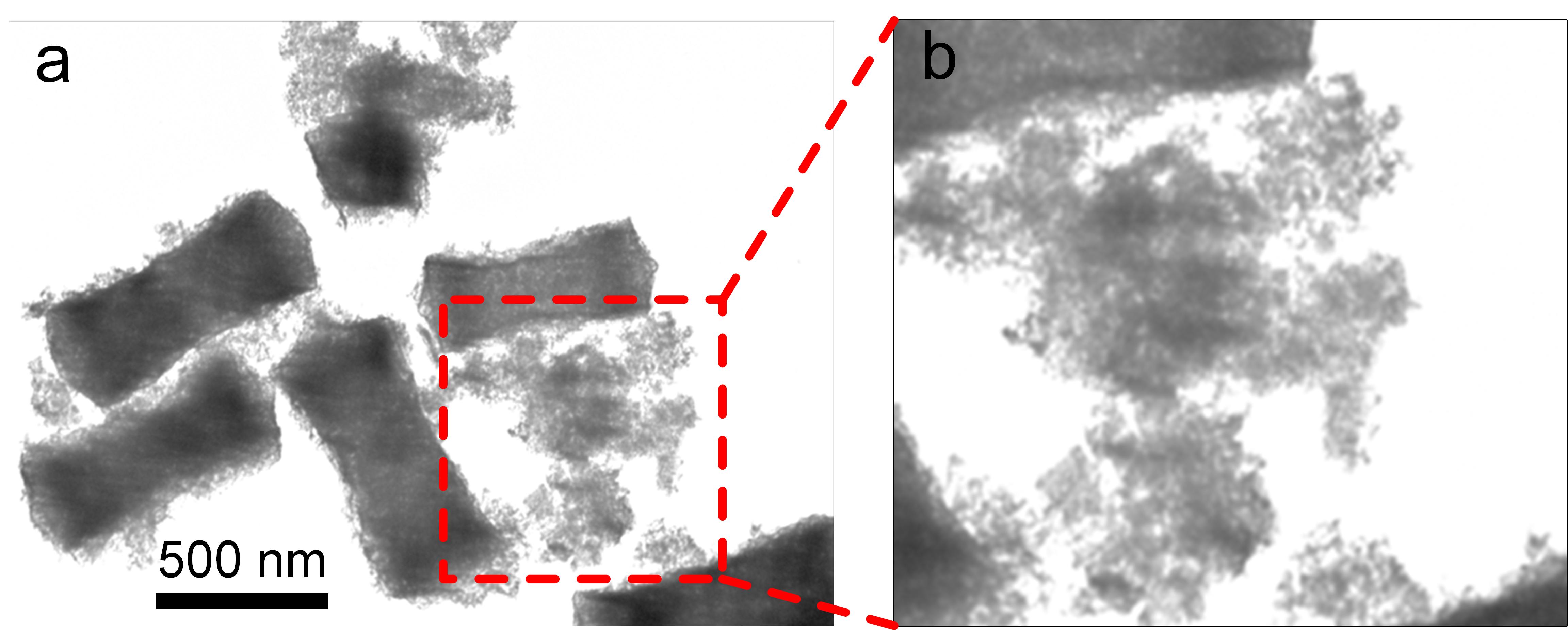


**Figure S6** TEM image of the product obtained with a reaction time of 2 h.


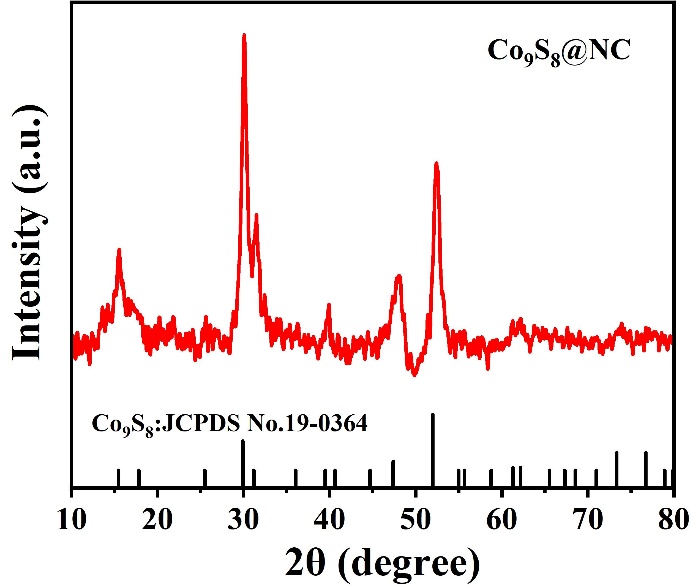


**Figure S7** XRD pattern of the as-synthesized Co_9_S_8_@NC sample.


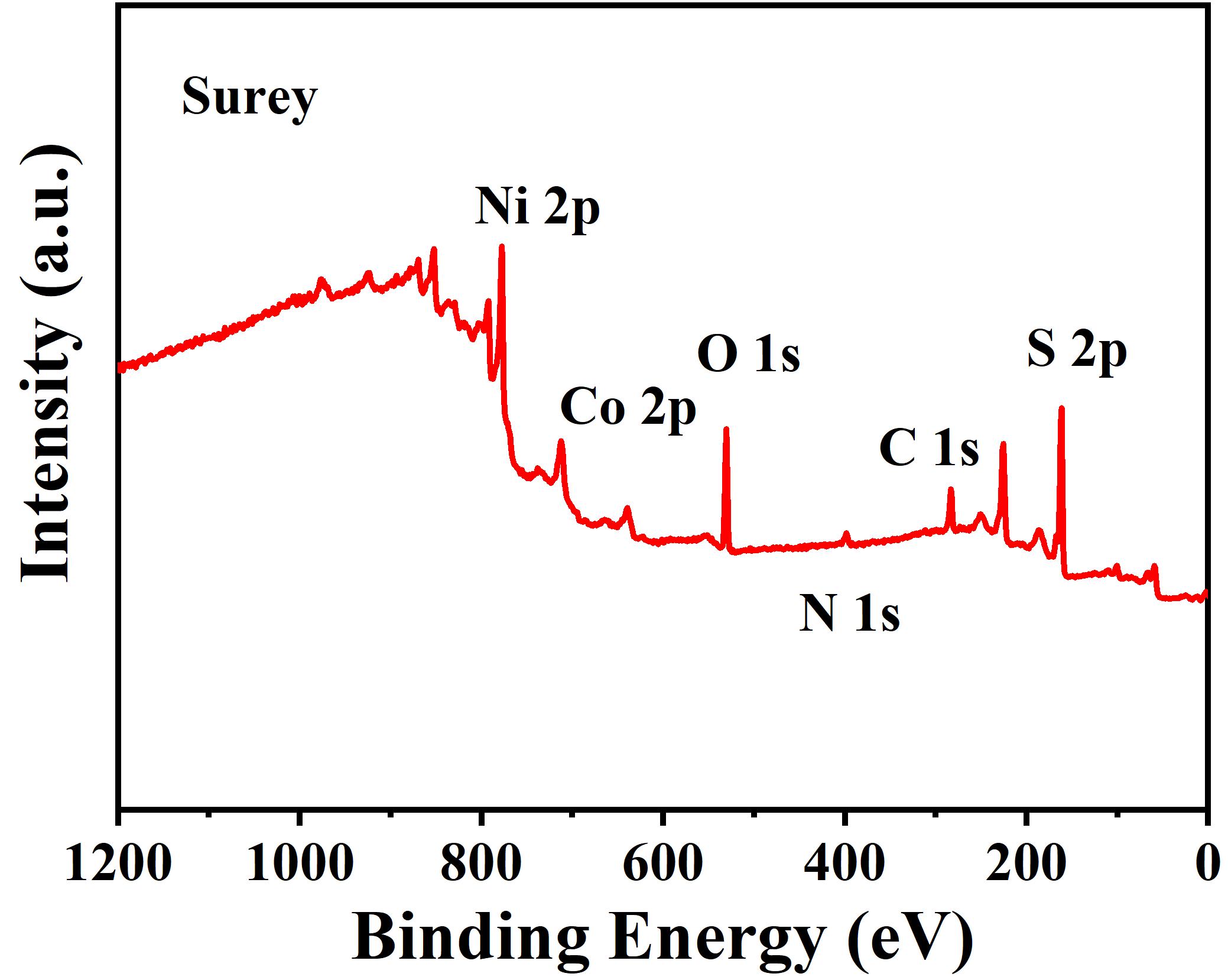


**Figure S8** The survey spectrum of the as-synthesized Ni_3_S_4_/Co_9_S_8_@NC.


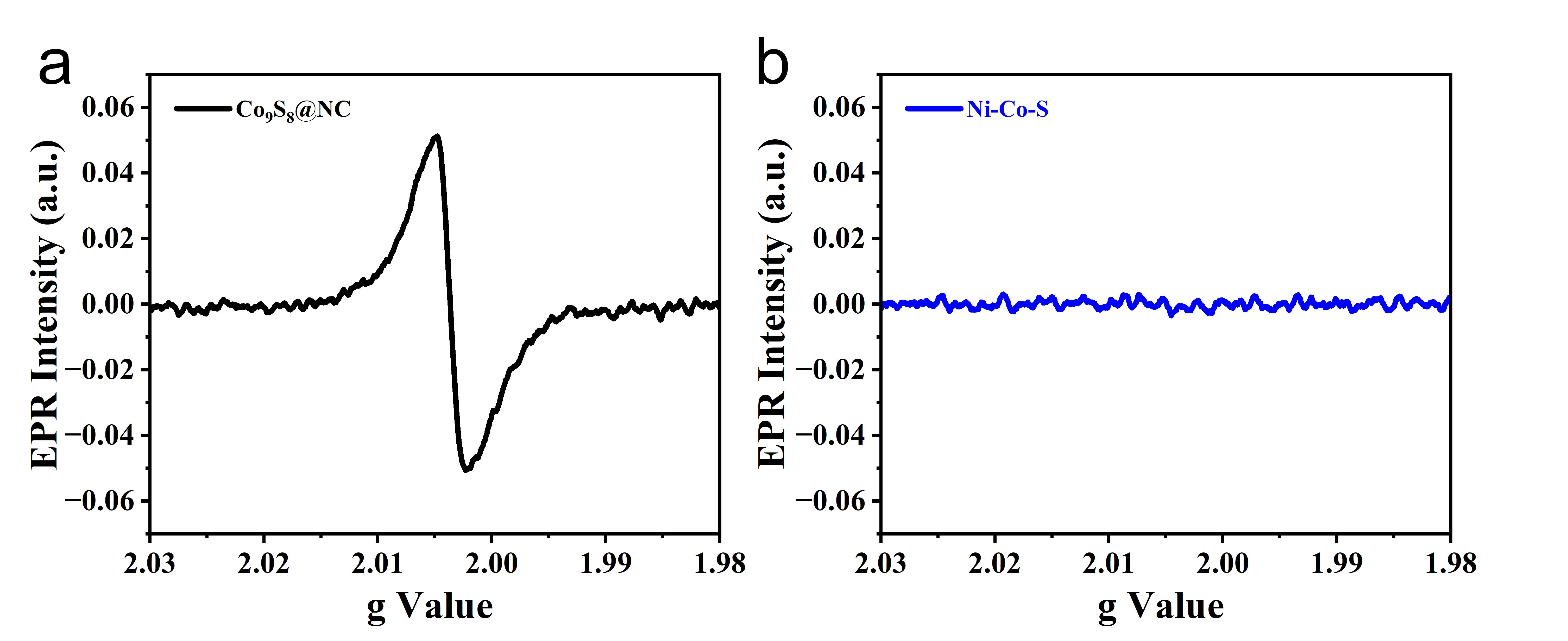


**Figure S9** ESR spectrum of the Co_9_S_8_@NC (a) and Ni-Co-S (b) sample.


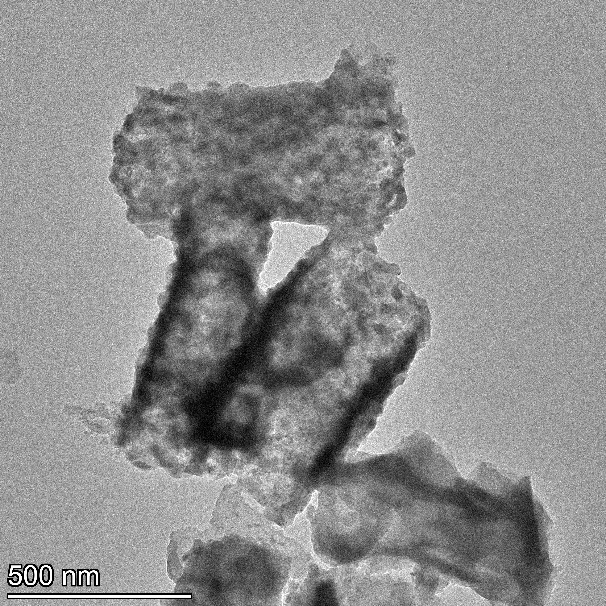


**Figure S10** The TEM image of the as-synthesized Co_9_S_8_@NC.


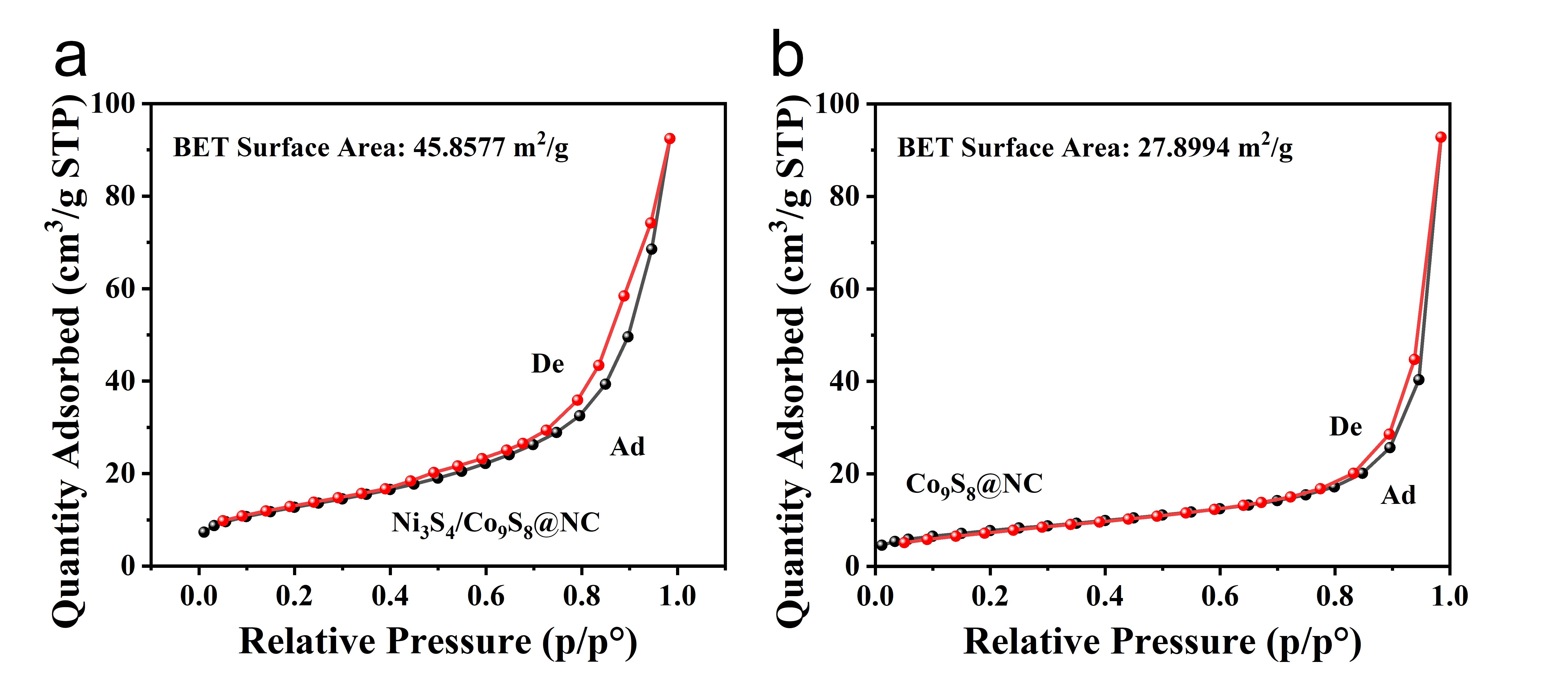


**Figure S11** BET surface area of the Ni_3_S_4_/Co_9_S_8_@NC and Co_9_S_8_@NC samples.


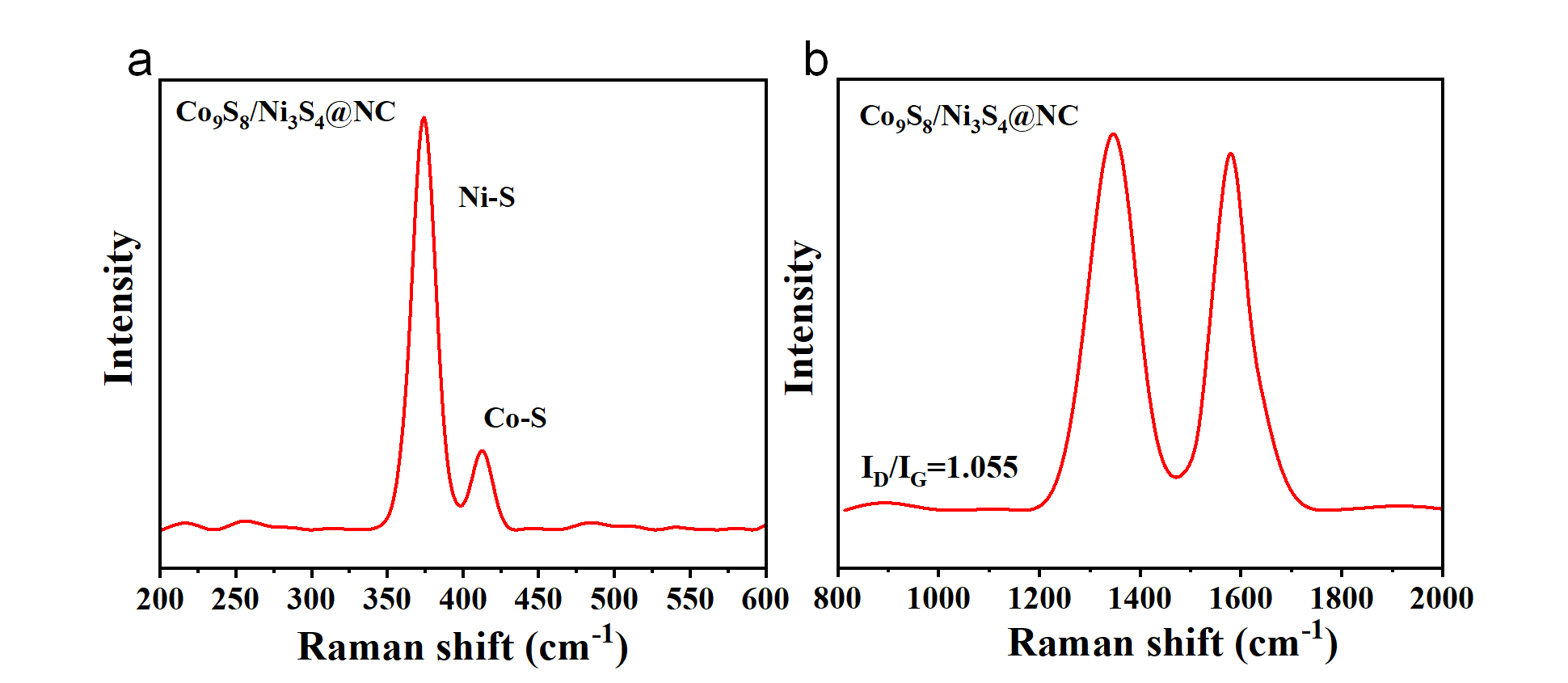


**Figure S12** The Raman spectrum of the Co_9_S_8_/Ni_3_S_4_@NC catalyst.

**Figure S13** The EDX spectrum of the Ni_3_S_4_/Co_9_S_8_@NC.


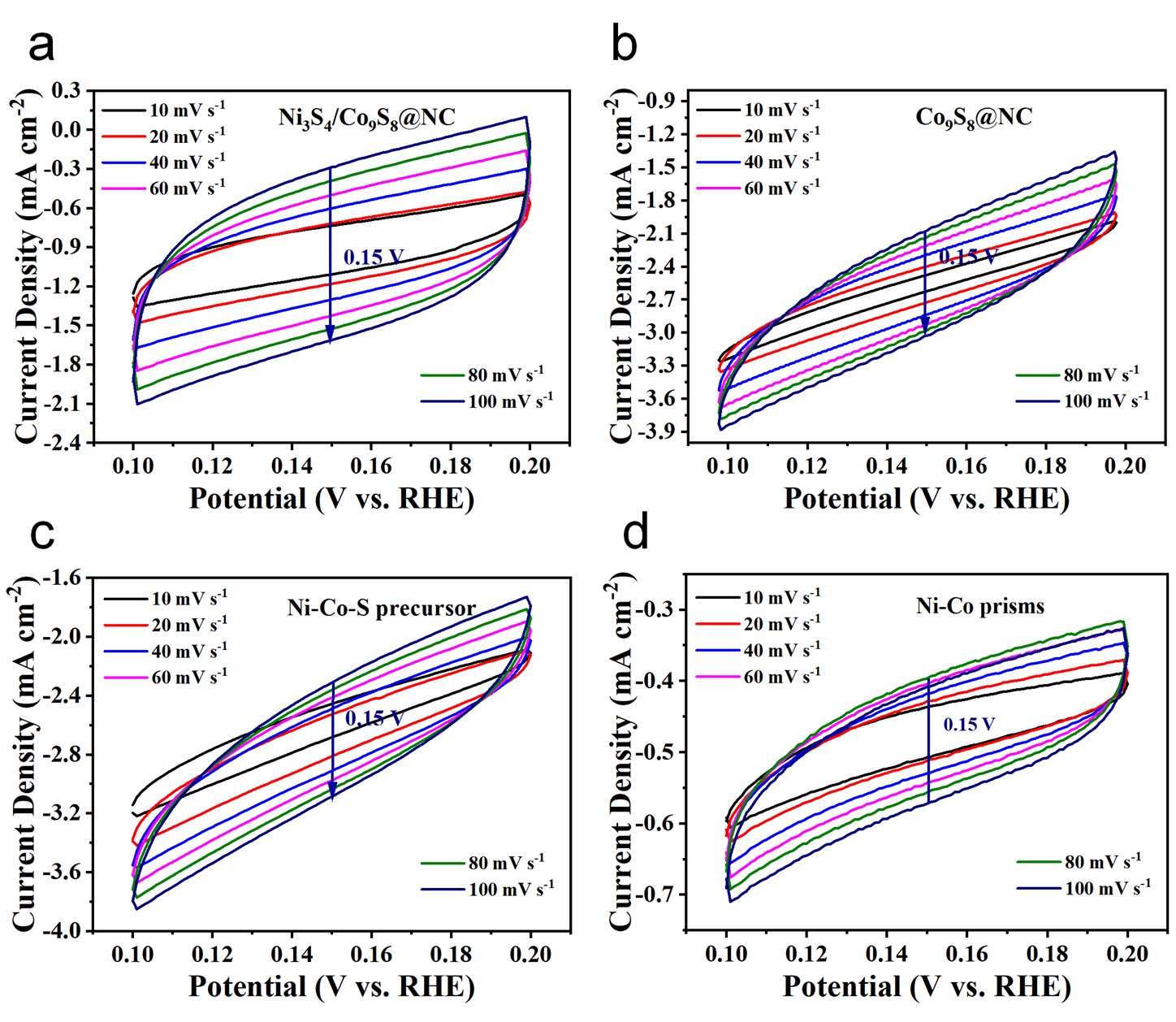


**Figure S14** Cyclic voltammetry of HER for the Ni_3_S_4_/Co_9_S_8_@NC (a), Co_9_S_8_@NC (b), Ni-Co-S (c) and Ni-Co prism (d) samples.


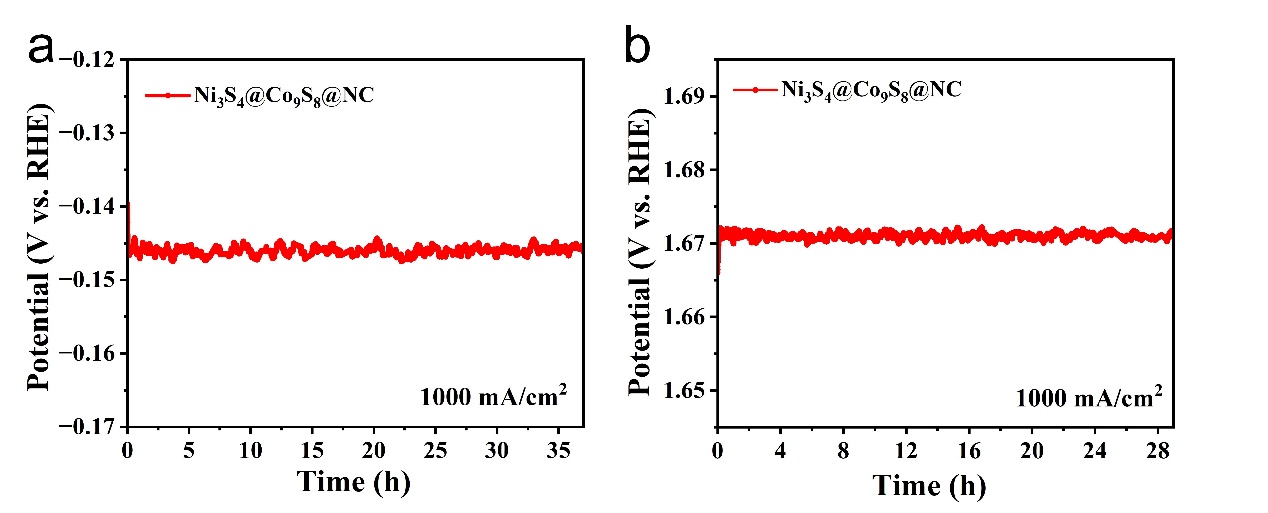


**Figure S15** The HER (a) and OER (b) stability test of the Co_9_S_8_/Ni_3_S_4_@NC catalyst under a current density of 1000 mA cm^–2^.


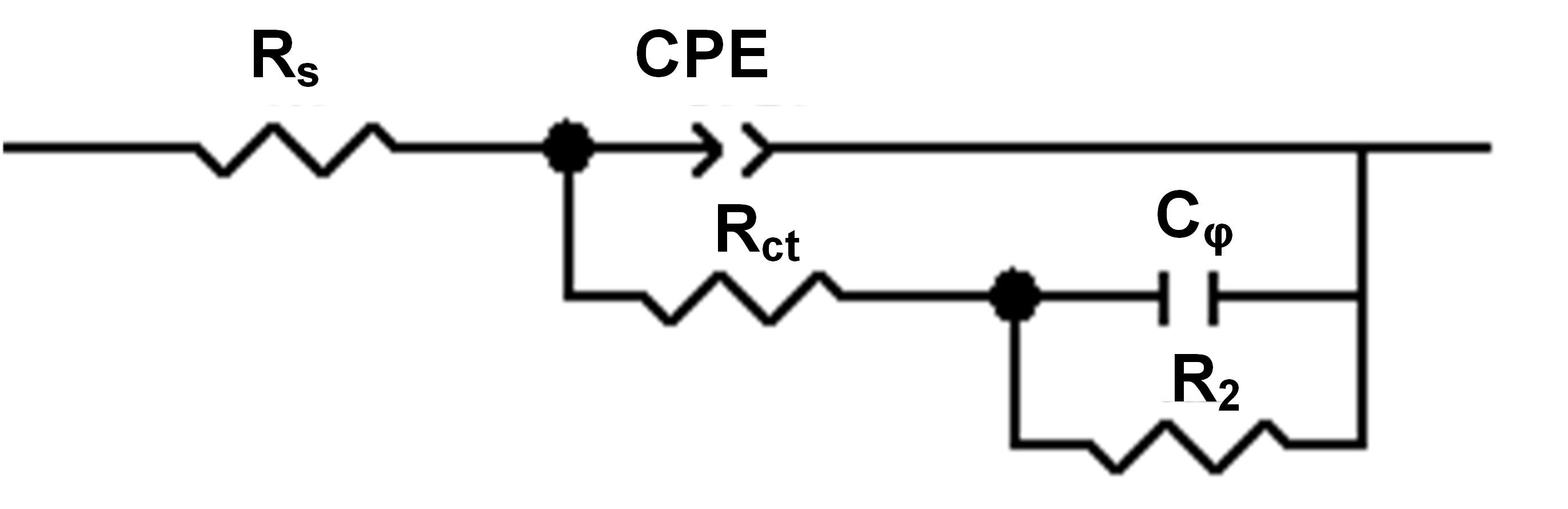


**Figure S16** The equivalent circuit of the in-situ EIS spectra.


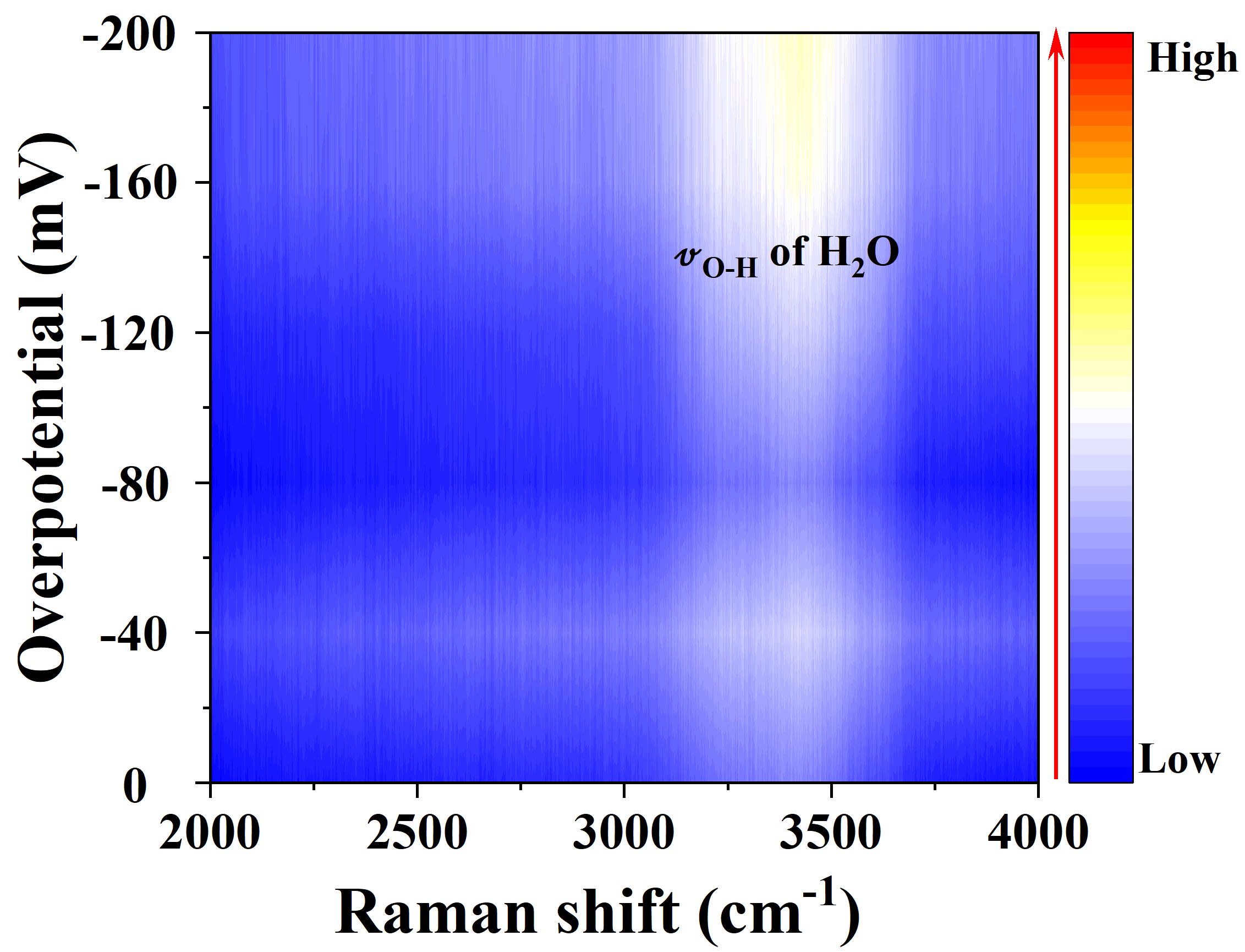


**Figure S17** 2D contour image of Co_9_S_8_@NC derived from in-suit Raman spectra.


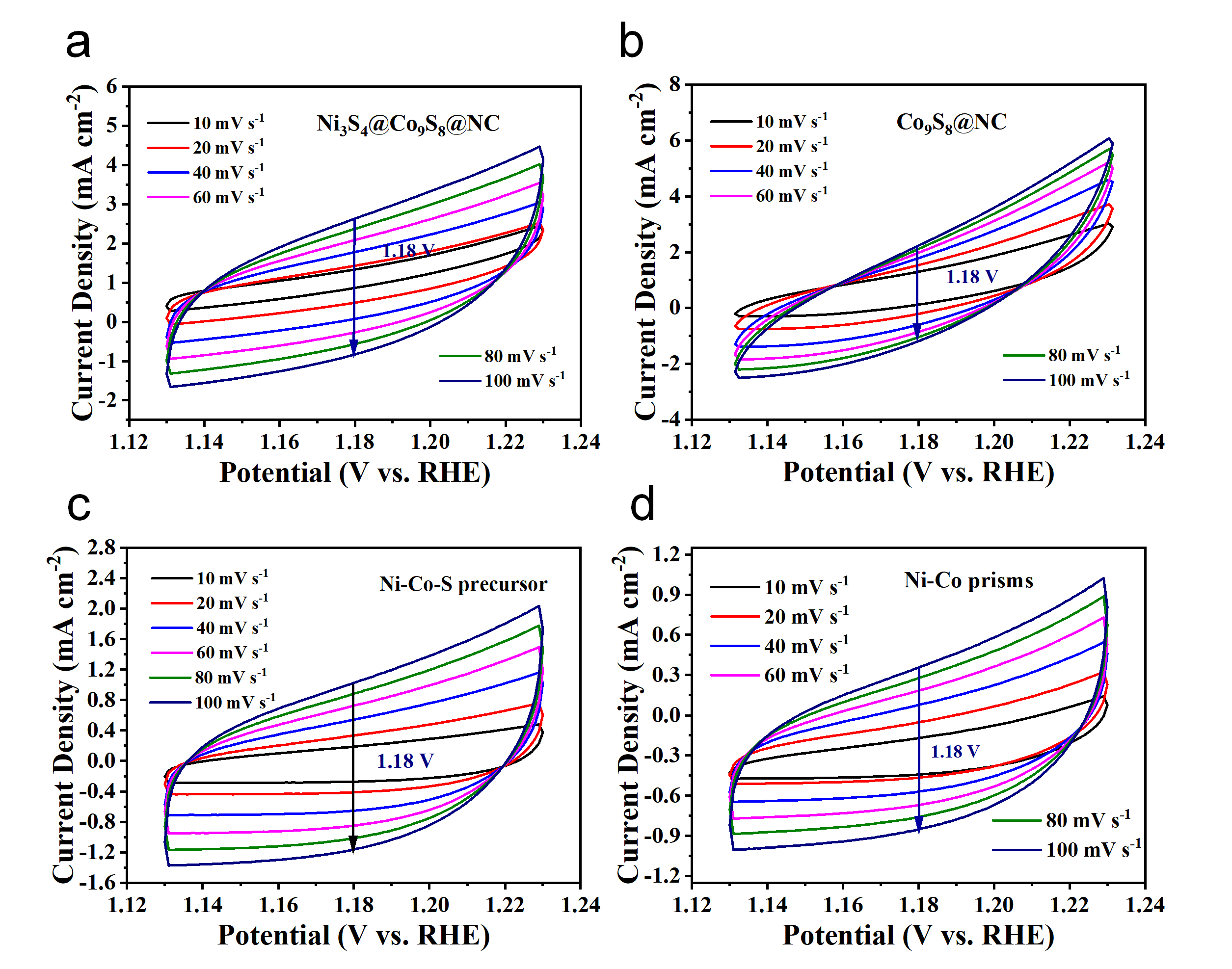


**Figure S18** Cyclic voltammetry of OER for the Ni_3_S_4_/Co_9_S_8_@NC (a), Co_9_S_8_@NC (b), Ni-Co-S (c) and Ni-Co prism (d) samples.


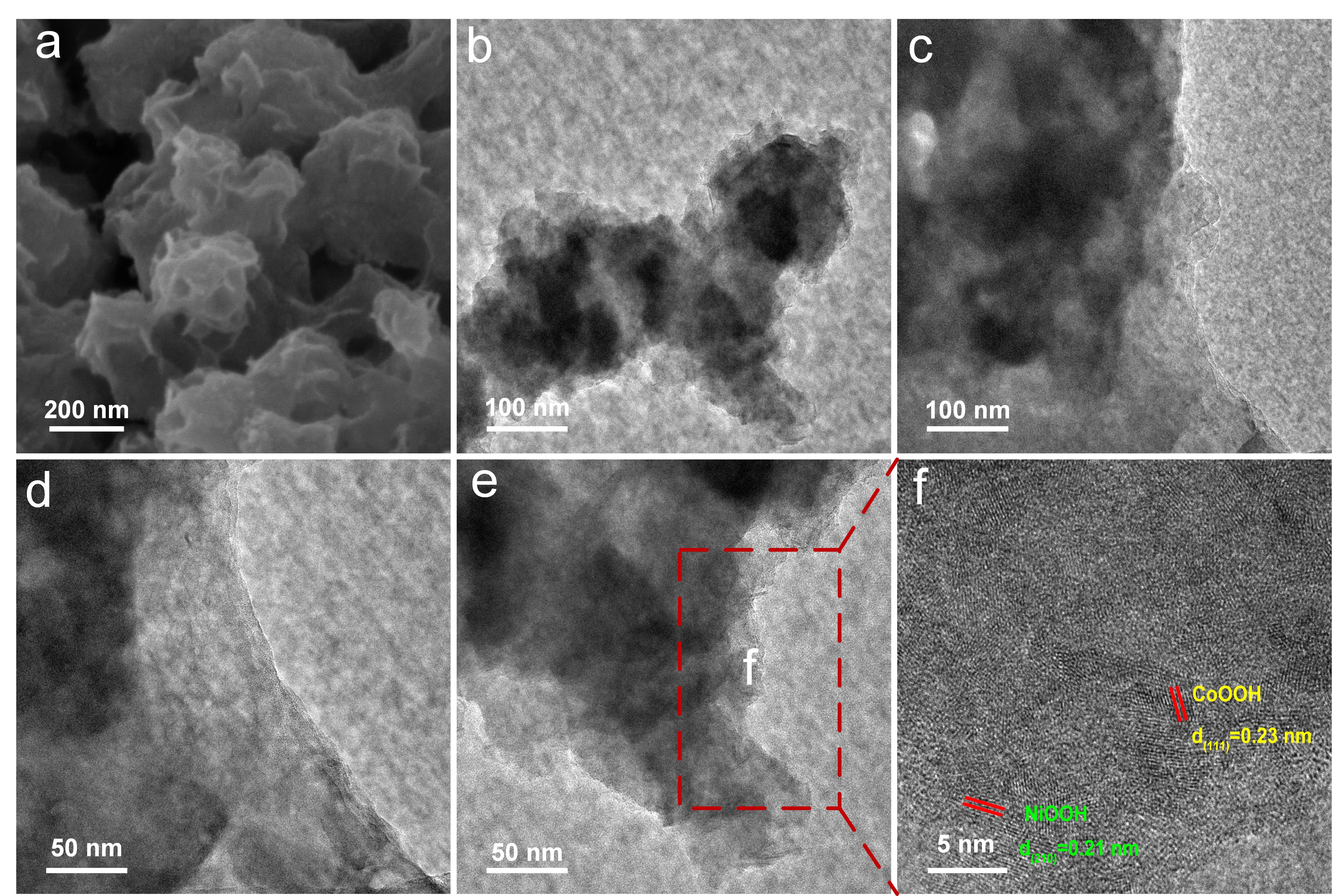


**Figure S19** SEM (a), TEM (b-e) and HRTEM (f) images of the Co_9_S_8_/Ni_3_S_4_@NC catalyst after OER stability test.


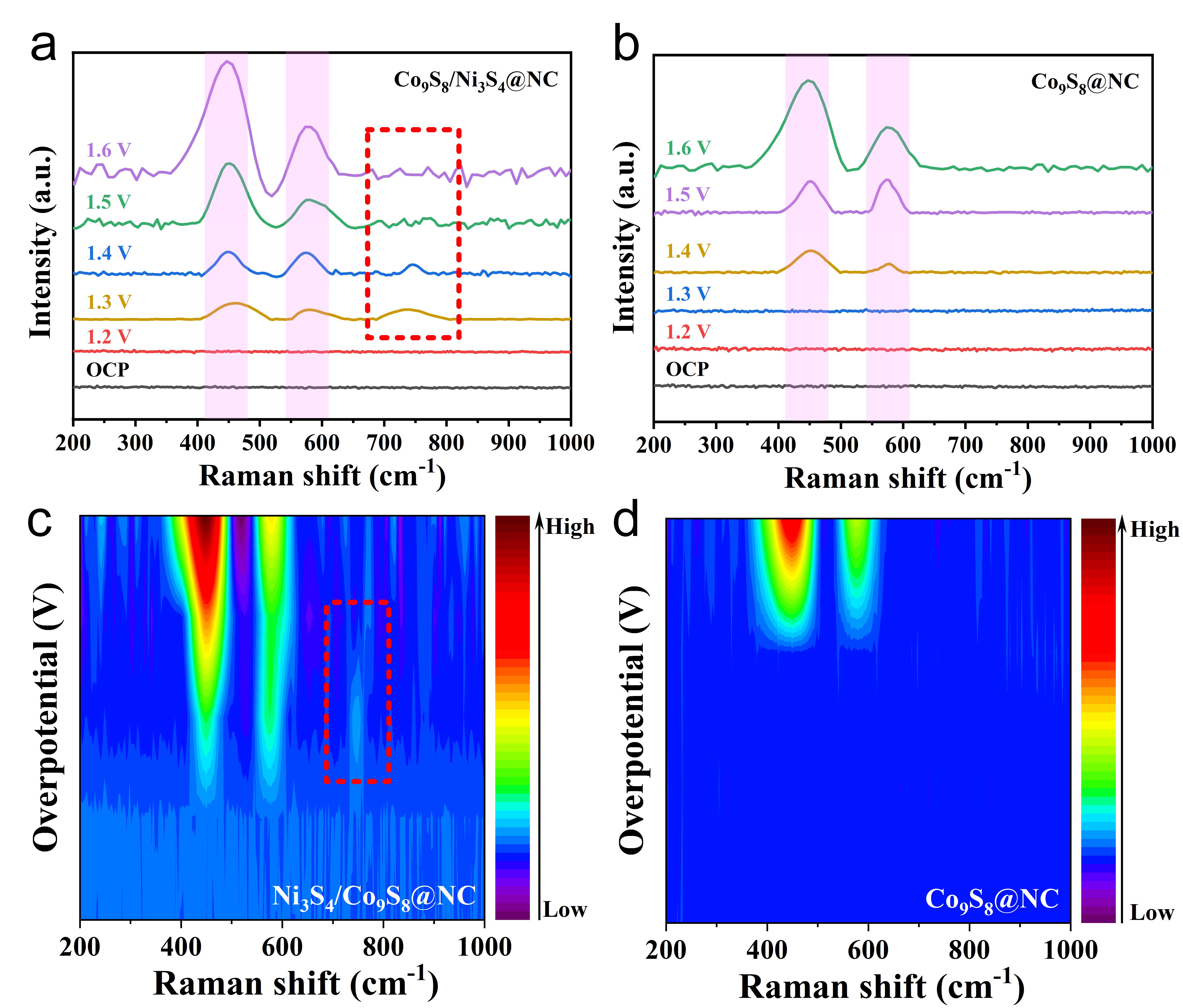


**Figure S20** In-situ Raman spectra and mappings of the as-synthesized Co_9_S_8_/Ni_3_S_4_@NC (a, c) and Co_9_S_8_/Ni_3_S_4_@NC and Co_9_S_8_@NC (b, d) in OER.


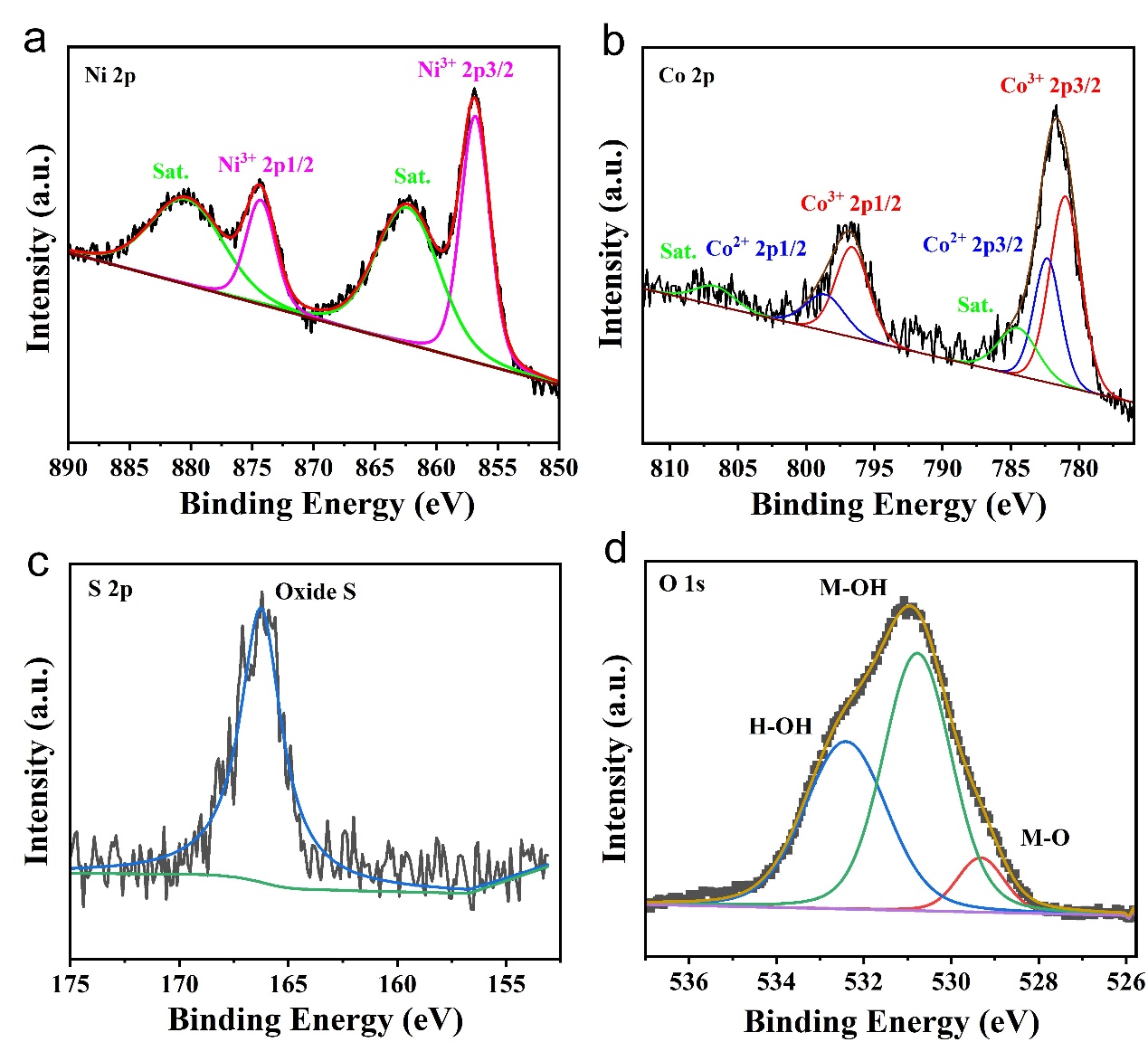


**Figure S21** XPS spectrum of the Co_9_S_8_/Ni_3_S_4_@NC after OER stability test.

| **Table S1**. EIS fitting data for Ni_3_S_4_/Co_9_S_8_@NC electrode in 1.0 M KOH | | | | | | |
| --- | --- | --- | --- | --- | --- | --- |
| Potential  (mV) | R_s_  (**Ω**) | CPE1  (F) | CPE2  (F) | R_ct_  (**Ω**) | C_1_  (μC) | R_2_  (**Ω**) |
| -5 | 6.677 | 0.032199 | 0.4951 | 8.019 | 0.032378 | 102.3 |
| -10 | 6.701 | 0.034322 | 0.4883 | 8.064 | 0.033027 | 95.1 |
| -15 | 6.632 | 0.030799 | 0.4712 | 8.211 | 0.035626 | 93.19 |
| -20 | 6.546 | 0.035762 | 0.47048 | 8.213 | 0.036527 | 72.92 |
| -25 | 6.544 | 0.037695 | 0.46758 | 8.434 | 0.037164 | 68.59 |
| -30 | 6.532 | 0.039295 | 0.4633 | 8.533 | 0.038766 | 62.51 |
| -35 | 6.511 | 0.040166 | 0.4536 | 8.61 | 0.039872 | 58.44 |
| -40 | 6.464 | 0.041766 | 0.4461 | 8.581 | 0.040462 | 53.12 |
| -45 | 6.417 | 0.041242 | 0.4433 | 8.612 | 0.041516 | 51.07 |
| -50 | 6.402 | 0.042166 | 0.4327 | 8.799 | 0.042341 | 49.22 |
| -55 | 6.404 | 0.043766 | 0.4219 | 8.803 | 0.043211 | 44.3 |

| **Table S2**. EIS fitting data for Co_9_S_8_@NC electrode in 1.0 M KOH | | | | | | |
| --- | --- | --- | --- | --- | --- | --- |
| **Potential**  **(mV)** | **R_s_**  **(Ω)** | **CPE1**  **(F)** | **CPE2**  **(F)** | **R_ct_**  **(Ω)** | **C_φ_**  **(μC)** | **R_2_**  **(Ω)** |
| -5 | 12.61 | 0.022615 | 0.5637 | 12.58 | 0.012252 | 198.56 |
| -10 | 12.58 | 0.02662 | 0.5743 | 12.06 | 0.013422 | 207.09 |
| -15 | 12.21 | 0.031077 | 0.5871 | 12.61 | 0.014333 | 210 |
| -20 | 12.06 | 0.031113 | 0.5946 | 11.8 | 0.015437 | 179.01 |
| -25 | 12.01 | 0.035112 | 0.6143 | 11.61 | 0.018804 | 178.45 |
| -30 | 11.8 | 0.040324 | 0.6299 | 11.43 | 0.019767 | 164.34 |
| -35 | 11.61 | 0.042762 | 0.6261 | 10.799 | 0.020544 | 149.23 |
| -40 | 11.533 | 0.053722 | 0.6193 | 11.533 | 0.021096 | 146.78 |
| -45 | 11.43 | 0.051243 | 0.6022 | 11.2 | 0.02178 | 135.1 |
| -50 | 11.2 | 0.062788 | 0.60148 | 12.21 | 0.021877 | 123.45 |
| -55 | 10.799 | 0.062312 | 0.59858 | 12.01 | 0.023064 | 120.98 |

**Table S3** Comparison of the overpotentials at 10 mA cm^−2^ and the corresponding Tafel slopes with other state-of-the-art electrocatalysts for HER.

| **Catalysts** | **Overpotential**  **(mV)** | **Current density**  **(mA cm^–2^ )** | **Tafel slope (mV/dec)** | **Ref.** |
| --- | --- | --- | --- | --- |
| **Ni_3_S_4_/Co_9_S_8_@NC** | **83** | **10** | **31.41** | **This work** |
| CoP/NCNHP | 115 | 10 | 53 | [1] |
| FeCo-FeCoP@C@NCCs | 91 | 10 | 58 | [2] |
| Ni-Co-P-H | 121 | 10 | 68 | [3] |
| Multishelled Ni_2_P | 98 | 10 | 86.4 | [4] |
| MoS_2_-ZnO-Ni | 129 | 10 | 78 | [5] |
| QA-DBH/ZnO | 112 | 10 | 67 | [6] |
| NiFeAu LDH | 89 | 10 | 34 | [7] |
| Mo-Ni_2_P | 81 | 10 | 53.4 | [8] |
| Pt_1_@Fe-N-C | 105 | 10 | 85 | [9] |
| 1L-MoS₂/FTO | 150 | 10 | 45 | [10] |
| 1%Fe/Co-600 | 202 | 10 | 125.1 | [11] |
| S-NiFe_2_O_4_/NF | 170 | 10 | 61 | [12] |
| Pt-SA/MoOx | 85 | 10 | 123 | [13] |
| NiFe LDH/Cu NW | 103 | 10 | 59 | [14] |
| CoP/NiCoP | 75 | 10 | 64 | [15] |

**Table S4** Comparison of the overpotentials at 10 mA cm^−2^ and the corresponding Tafel slopes with other state-of-the-art electrocatalysts for OER.

| **Catalysts** | **Overpotential**  **(mV)** | **Current density**  **(mA cm^–2^ )** | **Tafel slope (mV/dec)** | **Ref.** |
| --- | --- | --- | --- | --- |
| **Ni_3_S_4_/Co_9_S_8_@NC** | **265** | **10** | **88.1** | **This work** |
| NiO/NiCo_2_O_4_ Nanosheets | 290 | 10 | 94 | [16] |
| α-Ni(OH)_2_ Nanosheets | 331 | 10 | 105 | [17] |
| NiFe-LDH/NF | 302 | 10 | 110 | [18] |
| Co_3_O_4_ Nanowires | 340 | 10 | 92 | [19] |
| MnO_2_/Graphene Composite | 360 | 10 | 98 | [20] |
| NiS Nanosheets | 280 | 10 | 90 | [21] |
| Fe-Doped NiO Microsphere | 336 | 10 | 101 | [22] |
| CoMn-LDH Nanoarrays | 320 | 10 | 95 | [23] |
| NiCo_2_O_4_ Spinels | 350 | 10 | 89 | [24] |
| Carbon-Coated Co_3_O_4_ | 355 | 10 | 109 | [25] |
| NiMoN Nanowires | 275 | 10 | 96 | [26] |
| Co-Fe Prussian Blue Analogue | 367 | 10 | 112 | [27] |
| LaCoO_3_ Perovskite | 390 | 10 | 120 | [28] |
| NiSe Nanowires | 285 | 10 | 105 | [29] |
| CoO@N-doped Carbon | 310 | 10 | 87 | [30] |

**References**

[1] Pan Y, Sun K, Liu S, et al. Core-shell ZIF-8@ ZIF-67-derived CoP nanoparticle-embedded N-doped carbon nanotube hollow polyhedron for efficient overall water splitting. Journal of the American Chemical Society, 2018, 140(7): 2610-2618.

[2] Li Y, Li S, Hu J, et al. Hollow FeCo-FeCoP@ C nanocubes embedded in nitrogen-doped carbon nanocages for efficient overall water splitting. Journal of Energy Chemistry, 2021, 53: 1-8.

[3] Liu X, Deng S, Xiao D, et al. Hierarchical bimetallic Ni-Co-P microflowers with ultrathin nanosheet arrays for efficient hydrogen evolution reaction over all pH values. ACS applied materials & interfaces, 2019, 11(45): 42233-42242.

[4] Sun H, Xu X, Yan Z, et al. Porous multishelled Ni_2_P hollow microspheres as an active electrocatalyst for hydrogen and oxygen evolution. Chemistry of Materials, 2017, 29(19): 8539-8547.

[5] Xu L, Wang S. A novel hierarchical MoS_2_-ZnO-Ni electrocatalyst prepared by electrodeposition coupling with dealloying for hydrogen evolution reaction[J]. Journal of Electroanalytical Chemistry, 2018, 808: 173-179.

[6] Mousavi N, Ensafi A A, Mousaabadi K Z, et al. Synthesis of quinacridone derivative supported on ZnO hexagonal as a new electrocatalyst for hydrogen evolution reaction[J]. Journal of Electroanalytical Chemistry, 2023, 928: 117029.

[7] Li X P, Han W K, Xiao K, et al. Enhancing hydrogen evolution reaction through modulating electronic structure of self-supported NiFe LDH. Catalysis Science & Technology, 2020, 10(13): 4184-4190.

[8] Li Z, Huang M, Li J, et al. Large‐scale, controllable synthesis of ultrathin platinum diselenide ribbons for efficient electrocatalytic hydrogen evolution. Advanced Functional Materials, 2023, 33(28): 2300376.

[9] X Zeng X, Shui J, Liu X, et al. Single‐Atom to Single‐Atom Grafting of Pt_1_ onto Fe-N_4_ Center: Pt_1_@Fe-N-C Multifunctional Electrocatalyst with Significantly Enhanced Properties[J]. Advanced Energy Materials, 2018, 8(1): 1701345.

[10] Voiry D, Fullon R, Yang J, et al. The role of electronic coupling between substrate and 2D MoS_2_ nanosheets in electrocatalytic production of hydrogen. Nature materials, 2016, 15(9): 1003-1009.

[11] Tang T, Jiang W J, Niu S, et al. Electronic and morphological dual modulation of cobalt carbonate hydroxides by Mn doping toward highly efficient and stable bifunctional electrocatalysts for overall water splitting. Journal of the American Chemical Society, 2017, 139(24): 8320-8328.

[12] Liu J, Zhu D, Ling T, et al. S-NiFe_2_O_4_ ultra-small nanoparticle built nanosheets for efficient water splitting in alkaline and neutral pH. Nano Energy, 2017, 40: 264-273.

[13] Xu J, Zhang C, Liu H, et al. Amorphous MoO_X_-Stabilized single platinum atoms with ultrahigh mass activity for acidic hydrogen evolution. Nano Energy, 2020, 70: 104529.

[14] Cai Z, Li L, Zhang Y, et al. Amorphous nanocages of Cu‐Ni‐Fe hydr (oxy) oxide prepared by photocorrosion for highly efficient oxygen evolution. Angewandte Chemie, 2019, 131(13): 4233-4238.

[15] Boppella R, Tan J, Yang W, et al. Homologous CoP/NiCoP heterostructure on N‐doped carbon for highly efficient and pH‐universal hydrogen evolution electrocatalysis. Advanced Functional Materials, 2019, 29(6): 1807976.

[16] Liu G, Cheng Y, Qiu M, et al. Facilitating interface charge transfer via constructing NiO/NiCo_2_O_4_ heterostructure for oxygen evolution reaction under alkaline conditions. Journal of Colloid and Interface Science, 2023, 643: 214-222.

[17] Chen H, Yan J, Wu H, et al. One-pot fabrication of NiFe_2_O_4_ nanoparticles on α-Ni(OH)_2_ nanosheet for enhanced water oxidation. Journal of Power Sources, 2016, 324: 499-508.

[18] Sarfraz B, Bashir I, Rauf A. CuS/NiFe-LDH/NF as a bifunctional electrocatalyst for hydrogen evolution (HER) and oxygen evolution reactions (OER). Fuel, 2023, 337: 127253.

[19] Zhou W, Wu X J, Cao X, et al. Ni_3_S_2_ nanorods/Ni foam composite electrode with low overpotential for electrocatalytic oxygen evolution[J]. Energy & Environmental Science, 2013, 6(10): 2921-2924.

[20] Arshad N, Usman M, Adnan M, et al. Nanoengineering of NiO/MnO_2_/GO ternary composite for use in high-energy storage asymmetric supercapacitor and oxygen evolution reaction (OER). Nanomaterials, 2022, 13(1): 99.

[21] Feng L L, Yu G, Wu Y, et al. High-index faceted Ni_3_S_2_ nanosheet arrays as highly active and ultrastable electrocatalysts for water splitting[J]. Journal of the American Chemical Society, 2015, 137(44): 14023-14026.

[22] Pebley A C, Decolvenaere E, Pollock T M, et al. Oxygen evolution on Fe-doped NiO electrocatalysts deposited via microplasma. Nanoscale, 2017, 9(39): 15070-15082.

[23] Lu Z, Xu W, Zhu W, et al. Three-dimensional NiFe layered double hydroxide film for high-efficiency oxygen evolution reaction. Chemical communications, 2014, 50(49): 6479-6482.

[24] Li Z, Li B, Chen J, et al. Spinel NiCo_2_O_4_ 3D nanoflowers supported on graphene nanosheets as efficient electrocatalyst for oxygen evolution reaction. International Journal of Hydrogen Energy, 2019, 44(31): 16120-16131.

[25] Chen P, Xu K, Fang Z, et al. Metallic Co_4_N porous nanowire arrays activated by surface oxidation as electrocatalysts for the oxygen evolution reaction. Angewandte Chemie, 2015, 127(49): 14923-14927.

[26] Tang C, Cheng N, Pu Z, et al. NiSe nanowire film supported on nickel foam: an efficient and stable 3D bifunctional electrode for full water splitting. Angewandte Chemie, 2015, 127(32): 9483-9487.

[27] Ishizaki M, Fujii H, Toshima K, et al. Preparation of Co-Fe oxides immobilized on carbon paper using water-dispersible Prussian-blue analog nanoparticles and their oxygen evolution reaction (OER) catalytic activities. Inorganica Chimica Acta, 2020, 502: 119345.

[28] Liu K, Li J, Wang Q, et al. Designed synthesis of LaCoO_3_/N-doped reduced graphene oxide nanohybrid as an efficient bifunctional electrocatalyst for ORR and OER in alkaline medium. Journal of Alloys and Compounds, 2017, 725: 260-269.

[29] Hou Y, Lohe M R, Zhang J, et al. Vertically oriented cobalt selenide/NiFe layered-double-hydroxide nanosheets supported on exfoliated graphene foil: an efficient 3D electrode for overall water splitting. Energy & Environmental Science, 2016, 9(2): 478-483.

[30] Liu T, Asiri A M, Sun X. Electrodeposited Co-doped NiSe_2_ nanoparticles film: a good electrocatalyst for efficient water splitting. Nanoscale, 2016, 8(7): 3911-3915.
